# Memantine to Treat Social Impairment in Youths With Autism Spectrum Disorder: A Randomized Clinical Trial

**DOI:** 10.1001/jamanetworkopen.2025.34927

**Published:** 2025-10-01

**Authors:** Gagan Joshi, Atilla Gönenc, Maura DiSalvo, Stephen V. Faraone, Tolga Atilla Ceranoglu, Amy M. Yule, Mai Uchida, Christopher J. McDougle, Janet Wozniak

**Affiliations:** 1 Alan and Lorraine Bressler Clinical and Research Program for Autism Spectrum Disorder, Massachusetts General Hospital, Boston; 2 Department of Psychiatry, Harvard Medical School, Boston, Massachusetts; 3 Department of Psychiatry, Upstate Medical University, Syracuse, New York; 4 Department of Psychiatry, Boston University School of Medicine, Boston Medical Center, Boston, Massachusetts; 5 Lurie Center for Autism, Massachusetts General Hospital, Boston

## Abstract

**Importance:**

Pharmacologic interventions for addressing social impairments in autism spectrum disorder (ASD) are lacking. Proton magnetic resonance spectroscopy (1H-MRS) studies in individuals with ASD have documented altered glutamate levels in the pregenual anterior cingulate cortex (pgACC).

**Objectives:**

To evaluate the safety and efficacy of memantine for treating social impairments in youths with ASD and to explore pgACC glutamate levels as a potential biomarker for treatment response.

**Design, Setting, and Participants:**

This 12-week, placebo-controlled, double-blind, parallel-design randomized clinical trial was conducted between January 20, 2015, and July 11, 2018. The study population comprised youths aged 8 to 17 years with ASD without intellectual disability (IQ≥85) recruited from ambulatory psychiatry clinics at an academic institution. Age- and sex-matched healthy control participants provided reference data for pgACC glutamate levels. Data analysis was conducted between January 7, 2020, and December 19, 2024.

**Interventions:**

Participants with ASD were randomized to memantine or placebo, with dose titration up to 20 mg/d. 1H-MRS scans were acquired to assess pgACC glutamate levels.

**Main Outcomes and Measures:**

Response was defined a priori as (1) a 25% or greater reduction in informant-rated Social Responsiveness Scale–Second Edition total scores and (2) a clinician-rated Clinical Global Impression–Improvement subscale (anchored for ASD) score of 2 or less. The association between pgACC glutamate levels and treatment response was explored using receiver operating characteristic (ROC) curve analysis.

**Results:**

This study included 42 youths with ASD who initiated treatment (mean [SD] age, 13.2 [2.6] years; 32 males [76.2%]). Of these youths, 35 were included in the intention-to-treat efficacy analysis (n = 16 treated with memantine and 19 with placebo), and 33 completed the trial (n = 16 treated with memantine and 17 with placebo). Significantly more memantine-treated participants met the response criteria compared with placebo-treated participants (9 of 16 [56.2%] vs 4 of 19 [21.0%]; odds ratio, 4.8 [95% CI, 1.1-21.2]; *P* = .03). Memantine was well tolerated and did not have significantly more adverse events compared with placebo. Mean (SD) pgACC glutamate levels were significantly higher in youths with ASD vs healthy control participants (95.5 [14.6] IU vs 76.6 [17.7] IU; standardized mean difference, −1.2 [95% CI, −1.8 to −0.6]; *P* < .001). Abnormally elevated pgACC glutamate levels (≥1 SD above that of healthy control participants) were observed in 20 of 37 participants (54.0%) with ASD and were associated with more treatment responders to memantine than placebo (8 of 10 [80.0%] vs 2 of 10 [20.0%]; odds ratio, 16.0 [95% CI, 1.8-143.2]; *P* = .007). ROC curve analysis indicated that pgACC glutamate levels were highly efficient at identifying treatment responders.

**Conclusions and Relevance:**

In this trial, memantine was well tolerated and significantly improved social impairments in youths with ASD. Elevated pgACC glutamate levels were associated with a favorable treatment response, supporting their potential as a biomarker for assessing memantine efficacy in individuals with ASD.

**Trial Registration:**

ClinicalTrials.gov Identifier: NCT01972074

## Key Points

**Question:**

Does treatment with memantine improve social functioning in youths with autism spectrum disorder (ASD) without intellectual disability?

**Findings:**

In this randomized clinical trial that included 42 youths with ASD, memantine was well tolerated and significantly improved social impairments, particularly in youths with abnormally high glutamate levels in the pregenual anterior cingulate cortex region of the brain.

**Meaning:**

Memantine appears to be safe and effective for treating social impairments in patients with autism, particularly for those with glutamate dysregulation.

## Teaser

This randomized clinical trial evaluates the efficacy of memantine for the treatment of social impairments in youths with autism spectrum disorder.

## Introduction

Autism spectrum disorder (ASD) is characterized by deficits in social communication and interaction (SCI), along with restricted and repetitive behaviors (RRBs), affecting more than 2% of children in the general population.^1^ Despite its prevalence and impact, no medication has reliably demonstrated efficacy in improving social impairment in individuals with ASD.^2,3,4,5,6,7^

Glutamate, the primary excitatory neurotransmitter in the brain, modulates neuronal development and synaptic plasticity through *N*-methyl-d-aspartate (NMDA) receptor activity.^8^ Multiple lines of investigation, including serologic, postmortem brain, genetic, and proton magnetic resonance spectroscopy (1H-MRS) studies, have suggested that glutamate is dysregulated in individuals with ASD.^9,10,11,12,13,14,15,16^ The pregenual anterior cingulate cortex (pgACC), a brain region rich in glutamate neurotransmission, subserves social processing, emotional awareness, mentalizing, and self-reflection functions that are impaired in individuals with ASD.^17,18,19^ 1H-MRS meta-analyses note conflicting findings regarding alterations in glutamate concentration in individuals with ASD.^20,21,22,23,24,25^ However, prior studies in youths with ASD, including pilot work using 2-dimensional 1H-MRS at 4T,^25^ have documented elevated glutamate levels in the pgACC, suggesting that glutamatergic abnormalities may characterize a biologically meaningful subgroup with ASD relevant to treatment response.^20,21,22,23,24,25^

Several glutamate-modulating agents, including lamotrigine, amantadine, and *N*-acetylcysteine, have been evaluated as potential treatments for the core symptoms of ASD, demonstrating only modest efficacy.^26,27,28,29,30^ In contrast, preliminary data from retrospective and prospective uncontrolled trials of memantine hydrochloride, with its unique mechanism of action as a moderate-affinity noncompetitive NMDA receptor antagonist, have been promising, reporting an acceptable safety and tolerability profile and substantial improvements in SCI and RRBs in youths and adults with ASD.^31,32,33^ However, the only controlled trial of memantine in children to date, while showing improvements in ASD behaviors, failed to demonstrate superiority over an equally robust placebo response; this is likely due to the low dosing and inclusion of participants with intellectual disability, which did not adequately assess memantine’s efficacy in individuals with ASD without intellectual disability.^34^ Addressing these limitations, preliminary findings from an uncontrolled trial of memantine at dosages of up to 20 mg/d in adults with ASD without intellectual disability demonstrated substantial improvements in social behaviors.^35^

Collectively, the aforementioned studies suggest that glutamate-modulating agents could be beneficial for the core features of autism and that brain glutamate levels hold promise as a biomarker of interest for the treatment of ASD. This 12-week, parallel-design, double-blind randomized clinical trial (RCT) aimed to evaluate the short-term efficacy and tolerability of memantine for the treatment of social impairments in youths with ASD without intellectual disability. An exploratory aim was to examine the association between pgACC glutamate levels and response to memantine. Based on preliminary studies, we hypothesized that memantine would be well tolerated and effective, and that pgACC glutamate levels would be elevated in individuals with ASD relative to healthy control participants and associated with memantine response.^25,35^

## Methods

This parallel-design, double-blind RCT was conducted in compliance with the principles of the Good Clinical Practice guideline and was approved by the Institutional Review Board of Partners Healthcare, which oversees human participant research conducted by Massachusetts General Hospital (MGH) and McLean Hospital. Written informed consent was provided by the parent or legal guardian of each participant. Participants younger than 14 years and 14 years or older provided written informed assent and consent, respectively. The trial protocol is available in Supplement 1. This study followed the Consolidated Standards of Reporting Trials (CONSORT) reporting guideline.

Between January 20, 2015, and July 11, 2018, participants with ASD were recruited from ambulatory care referrals to a general child and adolescent psychiatry clinic and a specialized ASD clinic at an academic institution. Healthy control participants were recruited from the general population.

### Eligibility Criteria for Participants

Eligible participants with ASD were youths aged 8 to 17 years without intellectual disability whose symptoms met *Diagnostic and Statistical Manual of Mental Disorders*, *Fifth Edition*^36^ diagnostic criteria for autism, as established with a clinical diagnostic evaluation and having at least moderately severe ASD. Moderately severe ASD was assessed with the following: (1) an informant-rated Social Responsiveness Scale–Second Edition School-Age Form (SRS-2) total raw score of 85 or greater^37^ and (2) a clinician-rated Clinical Global Impression–Severity subscale (anchored for ASD [ASD-CGI-S]) score of 4 or greater.^38^ Healthy control participants were age, sex, and IQ matched with enrolled participants with ASD. Healthy control participants had no notable autistic traits (SRS-2 raw score <60) and no major psychopathology, as established with the Kiddie-Schedule for Affective Disorders and Schizophrenia–Epidemiologic Version^39^ semistructured interview and confirmed with a clinical diagnostic interview.

### Study Design, Randomization, and Intervention

This study was a 12-week, double-blind, parallel-design RCT in which youths with ASD were randomized 1:1 to receive memantine or placebo. An independent statistician created a computer-generated randomization schedule using block randomization with randomly selected block sizes of 4 and 6, stratified by sex (male vs female) and race and ethnicity. Based on the racial and ethnic distribution of the referral source population, 2 categories were established for race and ethnicity when creating the randomization schedule. Race and ethnicity were self-reported as White or other race or ethnicity; these data were included to serve as a basis for hypothesis generation that would justify further data collection on a large racial and ethnic minority subsample. The randomization schedule was provided to the MGH Clinical Trials Pharmacy for assignment. The study medication was titrated to a maximum daily dose of 20 mg over the first 4 weeks and then maintained at the maximum achieved dose until the end of the trial. Titration of the study medication was guided by a flexible titration schedule, which allowed for slower titration or maintenance at a lower dose based on tolerability per clinician judgment.

### Measures and Procedures

The efficacy response of memantine for the treatment of core features of ASD was assessed as the primary outcome with the informant-rated SRS-2 and the clinician-rated CGI–Improvement subscale (anchored for ASD [ASD-CGI-I]) with a priori criteria for treatment response defined as a 25% or greater improvement in the SRS-2 total raw score and an ASD-CGI-I score of 2 or less (indicating much improved or very much improved). Core features of autism were further examined with the SRS-2, the Social Withdrawal subscale of the Aberrant Behavior Checklist (ABC-SW),^40^ and the Children’s Yale-Brown Obsessive Compulsive Scale modified for pervasive developmental disorder.^41^ Treatment response of associated psychopathologies was assessed with the clinician-rated Attention Deficit Hyperactivity Disorder Rating Scale–IV,^42^ the Children’s Depression Rating Scale–Revised,^43^ and the Anxiety Subscale of the Child and Adolescent Symptom Inventory–5.^44^ Level of global functioning change with treatment was assessed with the clinician-rated Global Assessment of Functioning scale.^45^ Safety and tolerability were monitored by conducting a complete physical examination, performing a battery of tests (blood testing, urinalysis, and electrocardiograms) at baseline and end point, and recording spontaneously reported treatment-emergent adverse events (TEAEs) and obtaining vital signs (pulse, blood pressure, and weight) at each study visit.

As an exploratory outcome, glutamate levels in the pgACC region of the brain were assessed by acquiring 1H-MRS imaging prior to initiating treatment in participants with ASD and twice at 12-week intervals in healthy control participants. The proton spectra were acquired by applying a 2-dimensional J-resolved 1H-MRS protocol that allows for the isolated analysis of glutamate levels.^46^ For details, refer to the eMethods in Supplement 2.

### Sample Size Determination

Based on the previous pilot study by Joshi et al,^35^ we estimated a 65% response rate to memantine treatment. In a previous RCT of risperidone in children with autism,^47^ response rates were 69.4% and 11.5% in the treatment and placebo groups, respectively. Therefore, sample size calculations were based on response rates of 65% in the memantine group and 11.5% in the placebo group. An a priori sample size of 40 participants (n = 20 participants per group) was set to detect a significant difference at 2-sided *P* < .05 with 96% power. The final sample for responder analysis consisted of 16 participants in the memantine group and 19 participants in the placebo group, providing 94% power to detect significant differences.

### Statistical Analysis

Efficacy analyses used a modified intention-to-treat (ITT) set, including all randomized participants with at least 1 postbaseline assessment. Treatment response was analyzed using the Pearson χ^2^ test at a predefined significance level of .05. Changes from baseline to end point in secondary efficacy outcomes were analyzed using mixed-effects regression models with robust SEs to account for the repeated measures for each participant. Glutamate levels were categorized based on *z* scores with the population mean (SD) derived from glutamate level measurements of healthy control participants, stratifying *z* scores of 1 or greater as high, scores between less than 1 and greater than −1 as medium, and scores of −1 or less as low. Receiver operating characteristic (ROC) curve analyses were performed to determine the efficiency of pgACC glutamate levels at identifying treatment responders. All analyses, except for treatment response, were exploratory. Safety data were summarized descriptively by treatment group and compared using the Fisher exact or Pearson χ^2^ test. Safety analyses included all participants who received treatment. All analyses were performed using Stata, version 18 (StataCorp), and occurred between January 7, 2020, and December 19, 2024.^48^

## Results

### Participants

**Figure 1.**
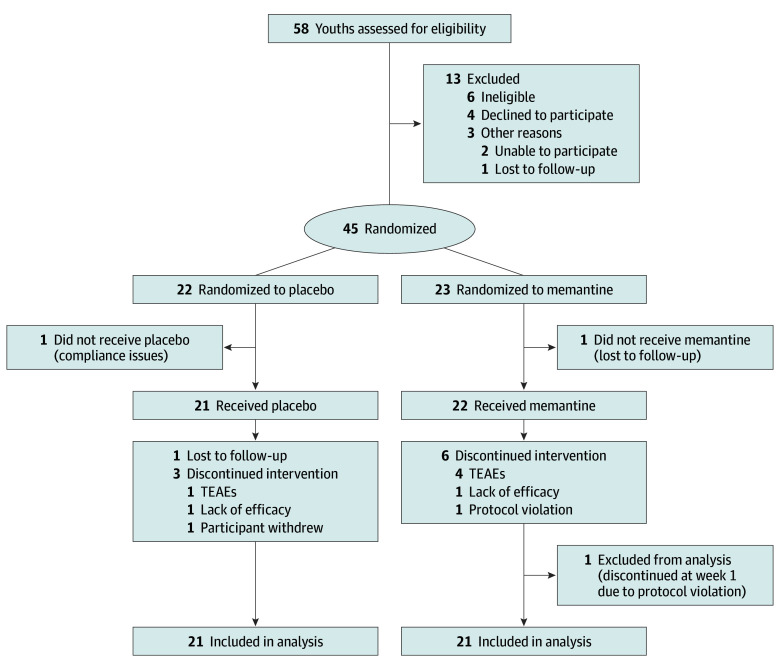
Participant Flow Diagram TEAE indicates treatment-emergent adverse event.

Of the 58 youths assessed for eligibility, 13 were excluded; the remaining 45 youths were randomized to treatment with either memantine (n = 23) or placebo (n = 22) (Figure 1). The major reasons for exclusion prior to randomization were ineligibility (n = 6), refusal to participate (n = 4), or loss to follow-up or inability to participate (n = 3). Of the 6 youths who were ineligible, 4 had subthreshold SRS scores (SRS raw score <85), 1 was actively using marijuana and alcohol, and 1 was experiencing psychiatric instability. Two participants (n = 1 in each group) were excluded prior to initiating trial medication. Participation of 1 additional memantine-treated youth was terminated in the first week of the trial due to protocol violation (initiated treatment with an exclusionary medication). Therefore, the final study sample comprised 42 youths (mean [SD] age, 13.2 [2.6] years; 32 males [76.2%] and 10 females [23.8%]), with 21 each in the memantine and placebo groups; 39 youths (92.9%) self-identified as White and 3 (7.1%) as other race or ethnicity. A total of 33 of 42 youths (78.6%) (16 of 21 [76.2%] in the memantine group and 17 of 21 [81.0%] in the placebo group) completed the trial (eTable 1 in Supplement 2).

Participants in both groups were comparable in terms of age, sex, race and ethnicity, and profiles of clinical characteristics, including psychiatric comorbidities and concomitant medications (Table 1). The modified ITT analysis of primary and secondary outcomes included 16 of 21 youths (76.2%) in the memantine group and youths 19 of 21 (90.5%) in the placebo group, totaling 35 youths altogether, who completed at least 1 repeat assessment during the trial.

**Table 1.** Demographic, Clinical, and Treatment Characteristics^a^

Characteristic	Total sample (N = 42)	Treatment group		pgACC glutamate levels (n = 36)		
		Memantine (n = 21)	Placebo (n = 21)	High (n = 20)	Medium (n = 16)	*P* value
**Demographic profile**						
Age, mean (SD) [range], y	13.2 (2.6) [8-18]	13.1 (2.8) [8-18]	13.3 (2.5) [8-17]	13.1 (2.4) [8-17]	13.8 (2.6) [9-18]	.45
Preadolescents (8-12 y)	15 (35.7)	8 (38.1)	7 (33.3)	7 (35.0)	4 (25.0)	.72
Adolescents (13-18 y)	27 (64.3)	13 (61.9)	14 (66.7)	13 (65.0)	12 (75.0)	.72
Sex						
Female	10 (23.8)	5 (23.8)	5 (23.8)	6 (30.0)	2 (12.5)	.21
Male	32 (76.2)	16 (76.2)	16 (76.2)	14 (70.0)	14 (87.5)	
Race and ethnicity						
White	39 (92.9)	19 (90.5)	20 (95.2)	19 (95.0)	15 (93.8)	>.99
Other race or ethnicity^b^	3 (7.1)	2 (9.5)	1 (4.8)	1 (5.0)	1 (6.2)	
IQ (full scale), mean (SD)	107.0 (18.3)	104.0 (15.3)	110.0 (20.8)	108.7 (15.1)	108.9 (19.7)	.97
Tanner stage≥III^c^	32/41 (78.0)	15/21 (71.4)	17/20 (85.0)	14/19 (73.7)	14 (87.5)	.42
Body weight, kg						
Mean (SD) [range]	60.6 (20.5) [23.8-102.1]	56.8 (19.0) [23.8-89.5]	64.3 (21.5) [31.2-102.1]	66.1 (22.5) [27.5-10.21]	58.2 (17.0) [31.2-97.2]	.25
Underweight^d^	1 (2.4)	1 (4.8)	0	0	0	NA
Overweight^d^	12 (28.6)	3 (14.3)	9 (42.9)	9 (45.0)	2 (12.5)	.07
**Autism profile**						
ADOS-2 diagnosis	37/38 (97.4)	17/17 (100)	20 (95.2)	17/18 (94.4)	15/15 (100)	>.99
Autism	27/38 (71.0)	11/17 (64.7)	16 (76.2)	12/18 (66.7)	10/15 (66.7)	>.99
ASD	10/38 (26.3)	6/17 (35.3)	4 (19.0)	5/18 (27.8)	5/15 (33.3)	>.99
SRS-2 raw score, mean (SD)						
SRS total	108.4 (21.0)	113.3 (21.0)	103.6 (17.9)	113.1 (21.8)	101.3 (17.9)	.09
SRS-SCI	87.6 (17.1)	92 (18.0)	83.2 (15.4)	90.6 (18.8)	82.8 (16.3)	.20
SRS-RRB	20.2 (4.8)	21 (5.5)	19.5 (4.1)	21.6 (5.1)	18.1 (4.0)	.03
ASD-CGI-S score ≥4	42 (100)	21 (100)	21 (100)	20 (100)	16 (100)	NA
**Psychopathologic profile**						
Psychiatric disorders (lifetime)^e^						
No major psychopathology	2 (4.8)	0	2 (9.5)	1 (5.0)	1 (6.2)	>.99
ADHD	34 (81.0)	18 (85.7)	16 (76.2)	16 (80.0)	13 (81.2)	>.99
Multiple anxiety disorders (≥2)	32 (76.2)	16 (76.2)	16 (76.2)	15 (75.0)	12 (75.0)	>.99
Major depression	23 (54.8)	11 (52.4)	12 (57.1)	15 (75.0)	7 (43.8)	.06
Mania	7 (16.7)	4 (19.0)	3 (14.3)	4 (20.0)	3 (18.8)	>.99
Psychosis	6 (14.3)	4 (19.0)	2 (9.5)	3 (15.0)	2 (12.5)	>.99
**Functional profile**						
GAF score, mean (SD)	53.8 (2.1)	53.6 (2.5)	54.0 (1.6)	53.7 (2.2)	54.1 (2.1)	.58
**Psychopharmacotherapy profile**						
Concomitant pharmacotherapy	30 (71.4)	14 (66.7)	16 (76.2)	15 (75.0)	12 (75.0)	>.99
No. of medications used, mean (SD) [range]	2.2 (0.9) [1-4]	2.3 (0.7) [1-4]	2.1 (1.0) [1-4]	2.2 (0.9) [1-4]	2.3 (0.8) [1-4]	.73
1	6/30 (20.0)	1/14 (7.1)	5/16 (31.2)	3/15 (20.0)	1/12 (8.3)	.61
2	15/30 (50.0)	9/14 (64.3)	6/16 (37.5)	7/15 (46.7)	8/12 (66.7)	.44
≥3	9/30 (30.0)	4/14 (28.6)	5/16 (31.2)	5/15 (33.3)	3/12 (25.0)	.70
Psychotropic medication class						
SSRIs	17/30 (56.7)	6/14 (42.9)	11/16 (68.8)	8/15 (53.3)	7/12 (58.3)	.80
Stimulants	14/30 (46.7)	8/14 (57.1)	6/16 (37.5)	7/15 (46.7)	6/12 (50.0)	.86
Atomoxetine	3/30 (10.0)	0	3/16 (18.8)	1/15 (6.7)	2/12 (16.7)	.41
α-2 Agonists (guanfacine, clonidine)	9/30 (30.0)	6/14 (42.9)	3/16 (18.8)	3/15 (20.0)	4/12 (33.3)	.43
Melatonin	7/30 (23.3)	4/14 (28.6)	3/16 (18.8)	4/15 (26.7)	1/12 (8.3)	.22
Buspirone	5/30 (16.7)	2/14 (14.3)	3/16 (18.8)	2/15 (13.3)	3/12 (25.0)	.44
Aripiprazole	3/30 (10.0)	2/14 (14.3)	1/16 (6.3)	3/15 (20.0)	0	.10
Clonazepam	2/30 (6.7)	1/14 (7.1)	1/16 (6.3)	2/15 (13.3)	0	.19
Amitriptyline	2/30 (6.7)	2/14 (14.3)	0	1/15 (6.7)	1/12 (8.3)	>.99
Trazodone	1/30 (3.3)	0	1/16 (6.3)	1/15 (6.7)	0	>.99
Diphenhydramine	1/30 (3.3)	0	1/16 (6.3)	1/15 (6.7)	0	>.99

Abbreviations: ADHD, attention-deficit/hyperactivity disorder; ADOS-2, Autism Diagnostic Observation Schedule–Second Edition; ASD, autism spectrum disorder; ASD-CGI-S, Clinical Global Impression–Severity subscale (anchored for ASD); GAF, Global Assessment of Functioning; IQ, intelligence quotient; NA, not applicable; pgACC, pregenual anterior cingulate cortex; SRS-2, Social Responsiveness Scale–Second Edition School-Age Form; SRS-RRB, SRS-2 Restricted Repetitive Behavior; SRS-SCI, SRS-2 Social Communication and Interaction; SSRI, selective serotonin reuptake inhibitor.

^a^ Unless indicated otherwise, values are presented as No. of youths or as No. (%)/Total No. (%) of youths.

^b^ Self-reported as 2 categories (White or other race or ethnicity) based on the racial and ethnic distribution of the referral source population.

^c^ Per the Petersen Pubertal Development Scale.

^d^ Per Centers for Disease Control and Prevention stature-for-age and weight-for-age growth charts with 5th and 95th percentile cutoffs.

^e^ Kiddie Schedule for Affective Disorders and Schizophrenia-Epidemiological Version.

### Primary Outcome

The memantine group had a significantly higher treatment response rate than the placebo group (9 of 16 [56.2%] vs 4 of 19 [21.0%]; odds ratio, 4.8 [95% CI, 1.1-21.2]; *P* = .03). The number needed to treat (NNT) statistic based on the treatment response criteria was 3.

### Secondary Outcomes

Although the memantine group showed nominally greater reductions in scores on autism-related measures (SRS-2, SRS-SCI, SRS-RRB, and ABC-SW) with moderate effect sizes, these were not significant (eTable 2 in Supplement 2). At study end point, a substantial (although not significant) percentage of youths in the memantine group had reductions of 10 points or greater in SRS-2 total T score compared with placebo (10 of 16 [62.5%] vs 8 of 19 [42.1%]; *P* = .23), SRS-2 total T scores of 65 or less (7 of 16 [43.8%] vs 4 of 19 [21.1%]; *P* = .15), and CGI-S scores of 3 or less (10 of 19 [52.6%] vs 7 of 21 [33.3%]; *P* = .22). Additionally, of youths whose symptoms responded to treatment, a greater (albeit not significant) percentage in the memantine group saw a 25% or greater decrease in SRS-2 scores at week 6 (study midpoint) compared with placebo (8 of 9 [88.9%] vs 2 of 4 [50.0%]; *P* = .13). No differences were observed between the groups in scores on scales assessing associated psychopathology or global functioning (SMD, 0.2 [95% CI, −0.5 to 0.8]) (eTable 2 in Supplement 2).

Safety data for all 42 treated participants (n = 21 per group) were analyzed. Both the memantine and placebo groups had a similar dosage distribution, with most participants receiving 20 mg/d. No significant differences were observed between groups in terms of mean number of adverse events (AEs), percentage of participants reporting AEs, frequency of reported AEs, or rate of participants needing rescue medications. TEAEs of mild or moderate severity, occurring in 2 or more memantine-treated participants and at more than twice the frequency of placebo, included headache, upper respiratory conditions (eg, cold, infection, or allergies), increased or decreased appetite, suicidal ideation, sedation, and abdominal discomfort. Severe AEs were documented in 2 memantine-treated participants: one youth had transient severe headaches in weeks 2 and 6, although the AE was not titration or treatment limiting; another youth experienced severe worsening of irritability and agitation in the first week at 5 mg/d, leading to early trial termination. Three additional memantine-treated participants withdrew due to worsening anxiety during the titration phase of the trial (1 youth in the first week at 2.5 mg/d and 2 youths in the fourth week at 20 mg/d). One participant terminated placebo treatment in the second week because of worsening irritability. One memantine-treated participant experienced titration-limiting dizziness, restricting the dosage to 15 mg/d; this participant was a treatment responder at trial completion. Clinically meaningful high blood pressure and weight gain were not more frequent in either group (Table 2). Twelve-week memantine treatment was not associated with abnormal results on electrocardiograms or hematologic parameters, including liver function.

**Table 2.** Treatment Tolerability Response^a^

Characteristic^b^	Memantine (n = 21)	Placebo (n = 21)	*P* value
Study medication dosage, mean (SD) [range], mg/d	18.2 (4.9) [2.5-20.0]	19.0 (3.0) [10.0-20.0]	.51
Participants taking maximum trial dose (20 mg/d)	18 (85.7)	19 (90.5)	>.99
No. of AEs, mean (SD)	2.2 (1.2)	2.1 (1.5)	.87
Participants reporting AEs	14 (66.7)	9 (42.9)	.12
Headache (most commonly reported)	5 (23.8)	2 (9.5)	.41
Mild to moderate AEs^c^			
Headache	4 (19.0)	2 (9.5)	.66
New	3 (14.3)	2 (9.5)	>.99
Worsening	1 (4.8)	0	>.99
Insomnia	3 (14.3)	2 (9.5)	>.99
Cold, infection, or allergy	3 (14.3)	1 (4.8)	.61
Increased appetite	3 (14.3)	0	.23
Decreased appetite	2 (9.5)	0	.49
Suicidal ideation	2 (9.5)	0	.49
Sedation	2 (9.5)	1 (4.8)	>.99
Fatigue	2 (9.5)	2 (9.5)	>.99
Anxious or worried (worsening)	2 (9.5)	2 (9.5)	>.99
Abdominal discomfort	2 (9.5)	1 (4.8)	>.99
Severe AEs	2 (9.5)	0	.49
Headache	1 (4.8)	0	>.99
Irritability plus agitation (worsening)	1 (4.8)	0	>.99
Serious AEs	0	0	NA
Titration-limiting AEs	1 (4.8)	0	>.99
Participants completed trial (12 wk)	16 (76.2)	17 (81.0)	>.99
Early terminations			
Total	5 (23.8)	4 (19.0)	>.99
Treatment-limiting AEs	4 (19.0)	1 (4.8)	.34
Early termination due to lack of efficacy	1 (4.8)	1 (4.8)	>.99
Abnormal vital signs			
Developed high blood pressure^d^	1 (4.8)	2 (9.5)	>.99
Significant weight gain (≥7% increase)	2 (9.5)	2 (9.5)	>.99
Rescue medications			
No. of participants	1 (4.8)	2 (9.5)	>.99
Profile of rescue medication received			
Diphenhydramine	1 (100)	0	NA
Melatonin	0	2 (100)	NA

Abbreviations: AE, adverse effect; NA, not applicable.

^a^ Unless indicated otherwise, values are presented as No. (%) of youths.

^b^ Reported AEs were judged likely related to the study medication.

^c^ Reported in more than 5% of youths in the memantine group.

^d^ Systolic blood pressure of 130 mm Hg or greater, diastolic blood pressure of 85 mm Hg or greater, or both.

### Exploratory Outcome

**Figure 2.**
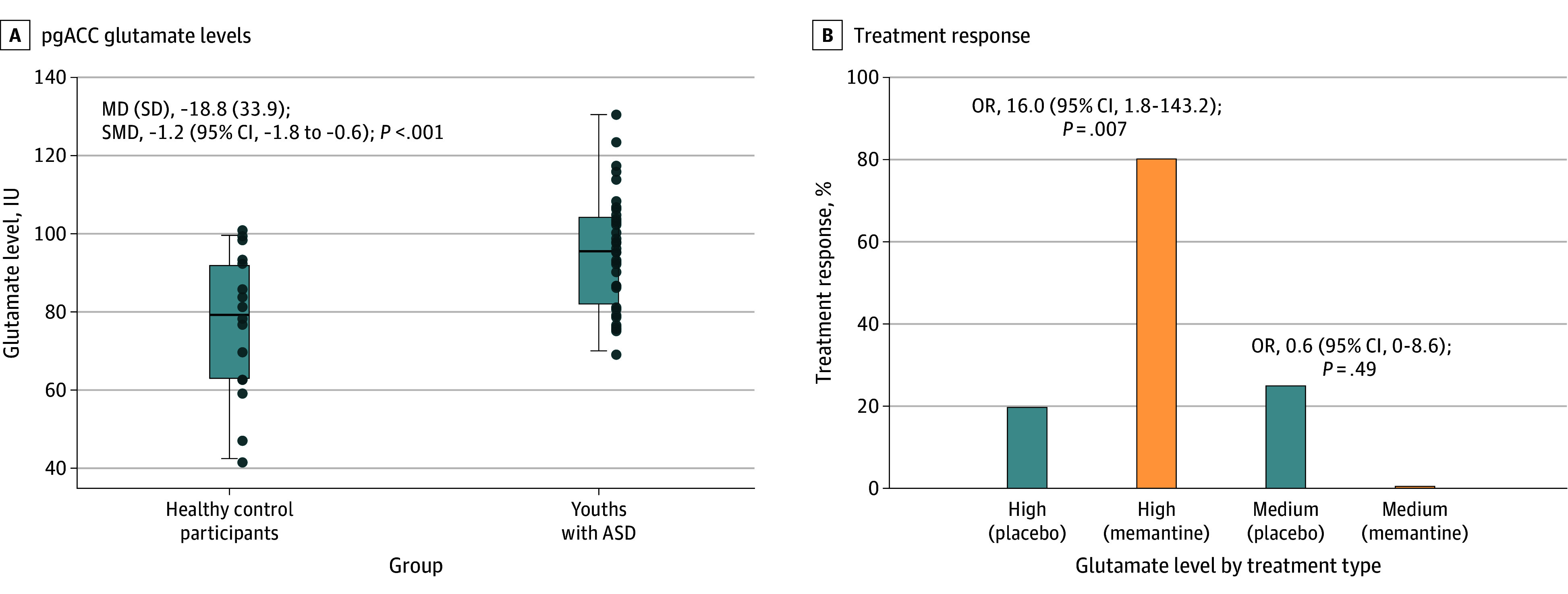
Treatment Response Based on Pregenual Anterior Cingulate Cortex (pgACC) Glutamate Levels Treatment response criteria were (1) a 25% or greater reduction in Social Responsiveness Scale–Second Edition School-Age Form score and (2) a Clinical Global Impression–Improvement subscale (anchored for autism spectrum disorder [ASD]) score of 2 or less. MD indicates mean difference; OR, odds ratio; SMD, standardized mean difference.

Spectroscopic pgACC glutamate data were collected for 37 participants with ASD and 16 healthy control participants. As illustrated in Figure 2A, pgACC glutamate levels were significantly higher in participants with ASD than in healthy control participants (95.5 [14.6] IU vs 76.6 [17.7] IU; mean [SD] difference, −18.8 [33.9]; standardized mean difference [SMD], −1.2 [95% CI, −1.8 to −0.6]; *P* < .001). The *z*-score mean (SD) of 76.5 (18.0) IU was derived from the 13 repeated measures and 3 single measures of glutamate levels in healthy control participants. Based on *z* scores, high pgACC glutamate levels (≥94.5 IU) were observed in 20 of 37 participants with ASD (54.0%), with the remaining 17 (46.0%) having medium levels (<94.5 IU and >58.5 IU). Abnormally elevated pgACC glutamate levels were associated with more treatment responders to memantine than placebo (8 of 10 [80.0%] vs 2 of 10 [20.0%]; odds ratio, 16.0 [95% CI, 1.8-143.2]; *P* = .007) (Figure 2B). The NNT improved from 3 in the unselected sample to 2 in the high-glutamate subsample.

**Figure 3.**
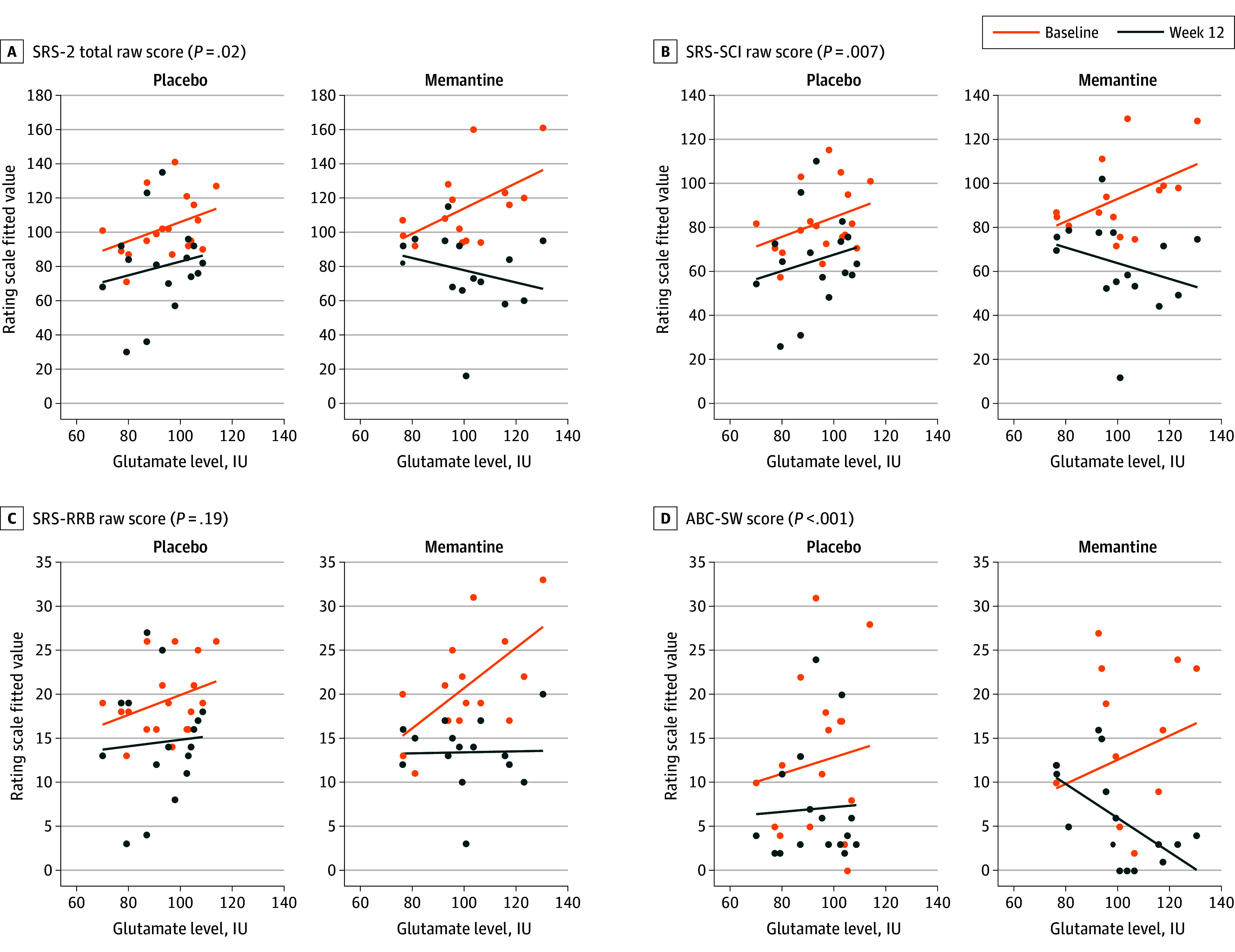
Treatment Response Based on Baseline Pregenual Anterior Cingulate Cortex (pgACC) Glutamate Levels ABC-SW indicates Aberrant Behavior Checklist-Social Withdrawal; SRS-2, Social Responsiveness Scale–Second Edition School-Age Form; SRS-RRB, SRS-2 Restricted Repetitive Behaviors; SRS-SCI, SRS-2 Social Communication and Interaction.

Figure 3 illustrates significant 3-way interactions between baseline pgACC glutamate levels, treatment group, and study visit when examining changes in SRS-2 total, SRS-SCI, and ABC-SW scores, but not SRS-RRB scores. Higher pgACC glutamate levels were associated with greater changes in SRS-2 total and SCI scores and ABC-SW scores in the memantine group, unlike in the placebo group. When stratified by pretreatment pgACC glutamate levels, effect sizes for all primary and secondary outcomes favored memantine in youths with ASD and high glutamate levels and placebo in youths with ASD and medium glutamate levels (eTable 3 in Supplement 2). The response rate to memantine was significantly higher than placebo in youths with ASD with high glutamate levels, but not in youths with ASD with medium glutamate levels (Figure 2B). The eFigure in Supplement 2 shows a significant difference in the area under the curve comparing the efficiency of pretreatment pgACC glutamate levels in identifying treatment response between the memantine and placebo groups (n = 15 vs 18; 0.93 [95% CI, 0.80-1.00] vs 0.34 [95% CI, 0.02-0.66]; *P* < .001). We identified an optimal pgACC glutamate level cutoff of 99 IU for memantine and 97 IU for placebo (eFigure in Supplement 2). The sensitivity, specificity, positive predictive power, and negative predictive power of this threshold were high and clinically actionable. Additional findings are presented in the eResults in Supplement 2.

## Discussion

The primary results of this 12-week RCT indicate that memantine was well tolerated and significantly more efficacious than placebo in improving social functioning in youths with ASD. Exploratory 1H-MRS analysis revealed abnormally elevated glutamate levels in the pgACC of participants with ASD relative to healthy control participants, with high efficiency at identifying responders to treatment with memantine.

In this study, treatment with memantine was superior to placebo in improving social behaviors. Youths who received memantine had 4.8 times the odds (95% CI, 1.1-21.2) of responding to treatment compared with placebo. The NNT statistic was robust (NNT = 3), indicating that 1 in 3 memantine-treated youths with ASD would respond to treatment. The markedly low NNT is particularly noteworthy in the context of ASD, in which effective treatments for core symptoms can be challenging to identify. This finding also suggests that memantine could be a relatively efficient treatment option, potentially leading to improved outcomes for a substantial proportion of patients, with minimal unnecessary exposure to those who may not benefit.

Further support for memantine’s potential clinical effectiveness in addressing core ASD features can be derived from the moderate effect sizes observed in secondary ASD outcome measures (SRS total, SRS-SCI, SRS-RRB, or ABC-SW scores) in this study (eTable 2 in Supplement 2). Nearly all of the memantine treatment responders (8 of 9 [88.9%]) met the a priori response criterion by week 6 of the trial. At 12-week trial completion, more than half of the participants saw clinically significant reductions in social deficit severity (SRS-2 total T score decrease of ≥10) and approximately half exhibited minimal to mild autism symptom severity (SRS-2 total T score ≤65 or CGI-S score ≤3).

The absence of meaningful response in measures assessing ASD-associated psychopathologies (attention-deficit/hyperactivity disorder, anxiety, or depression; eTable 2 in Supplement 2) suggests that improvements in social functioning cannot be attributed to these associated conditions. Furthermore, memantine showed no significant difference from placebo in global functioning measures, with a very small effect size (SMD, 0.2 [95% CI, −0.5 to 0.8]). Possible explanations for the lack of improvement in global functioning include a potential lag in the improvements in various indices of social functioning expected with the corresponding improvement in social behaviors, and they may reflect impairments related to associated psychopathologies that did not respond to treatment.

Spectroscopic glutamate levels in the pgACC were significantly elevated by a large magnitude in youths with ASD compared with healthy control participants, replicating previous findings by Joshi et al^25^ of glutamate dysregulation in individuals with ASD. Notably, the abnormally high levels of glutamate were not universal but were limited to 54.0% (n = 20 of 37) of participants with ASD, with the remainder of participants without any glutamate abnormality. As noted in Table 1, the study identified no specific clinical marker to distinguish individuals with ASD with high pgACC glutamate levels from those with medium levels.

Treatment response differed based on pgACC glutamate levels in participants with ASD. A significantly greater response rate to memantine compared with placebo was observed in the high-glutamate subsample, whereas no such difference was observed in the medium-glutamate subsample. All memantine responders had high glutamate levels, and the majority of participants with ASD with high glutamate levels were memantine responders (8 of 10 [80.0%]). The efficacy of memantine was more pronounced in youths with ASD and high glutamate levels across all outcome measures, whereas memantine performed worse than placebo in the medium-glutamate subsample (eTable 3 in Supplement 2). The NNT improved from 3 in the unselected sample to 2 in the high-glutamate subsample.

These findings suggest a selective response to memantine based on pgACC glutamate levels in youths with ASD. When baseline glutamate levels were taken into account, higher glutamate levels were associated with a significantly greater response to memantine with SRS-2 score as a continuous measure compared with placebo (Figure 3). This result suggests a promising role of pgACC glutamate levels to serve as a biomarker for identifying youths who may respond to memantine treatment. The levels of pgACC glutamate concentration demonstrated high efficiency at identifying responders to memantine treatment, which was significantly greater than its efficiency at identifying responders to placebo treatment (eFigure in Supplement 2). This marked difference indicates that the levels of pgACC glutamate concentration are efficient at identifying response to memantine but not placebo. ROC curve analysis identified a pgACC glutamate level of 99 IU as the optimal threshold for identifying memantine responders. The sensitivity, specificity, positive predictive power, and negative predictive power of this threshold were high and clinically actionable (eFigure in Supplement 2). Notably, this ROC-derived threshold (99 IU) was higher than the cutoff for abnormally high glutamate levels (≥94.5 IU) established from the normative range of healthy control participants. This finding indicates that the therapeutic response was associated with abnormally high pgACC glutamate levels in participants with ASD.

The efficacy responses in this study are both consistent with and divergent from the only previously reported 12-week RCT of memantine in children with ASD.^34^ When analyzing SRS-2 score as a continuous measure, both trials observed substantial improvements in ASD behaviors in both the memantine and placebo groups, with no statistically significant difference between groups. Although the profiles of change in mean SRS-2 scores were comparable in both trials, the effect size in the current trial was more robust. Additionally, this study assessed categorical treatment response as the primary outcome measure on which a significant difference was observed in the percentage of treatment responders, with results favoring the use of memantine. The discrepancies between the present trial and that by Aman et al^34^ may be attributed to several factors, including use of a substantially lower dose of memantine and the inclusion of children with ASD and comorbid intellectual disability in the prior trial. A key distinguishing feature of the current trial, which yielded the most notable findings, was the incorporation of glutamate levels into the analysis. Among participants with high glutamate levels, those receiving memantine exhibited clinically significant improvement in the continuous measure of SRS-2 scores compared with those receiving placebo, whereas there was no significant difference between the 2 treatment groups in participants with medium glutamate levels (eTable 3 in Supplement 2).

The findings of this trial suggest that treatment with memantine was very well tolerated. In general, AEs were transient and considered mild or moderate. The most frequently reported AEs in the memantine group were headaches, insomnia, upper respiratory conditions (cold, infection, or allergies), and increased appetite (Table 2). Notably, no participants experienced serious AEs. The tolerability profile is consistent with the trial by Aman et al,^34^ with upper respiratory tract symptoms and insomnia as the most common TEAEs and all treatment-limiting AEs occurring during the titration phase of the trial. Higher frequencies of irritability, aggression, and agitation with memantine than placebo treatment in the Aman trial contrast with the lower rates of irritability with memantine than placebo treatment in this study (4.8% vs 14%) (Table 2). The lower rate of irritability observed with memantine in the population of the present trial compared with the population in the trial by Aman et al^34^ may be attributable to the older age and absence of intellectual disability among participants

### Limitations

The results of this study should be interpreted in light of several methodologic limitations. When calculating the sample size, the placebo response rate was estimated from the largest treatment trial in youths with ASD examining the effects of risperidone on irritability because at the time there were no published RCTs of memantine in individuals with ASD. Although a concerted effort was made to recruit female participants, the final sample was predominantly male and was not powered to detect sex differences in treatment response. Because the study sample was predominantly White, the findings may not be generalizable to other racial and ethnic groups. Additionally, the study population of ASD youths was without intellectual disability, which may limit the generalizability of the results to broader populations with ASD. Due to the lack of well-established clinician-rated measures beyond the ASD-CGI, the treatment response of autism was assessed by outcome measures that relied solely on caretaker reporting. Although formal interrater reliability for the ASD-CGI ratings among trial investigators has not been established, low reliability would introduce random error, thereby reducing statistical power rather than biasing the results toward statistical significance. The sample of participants who terminated the trial early compared with those who completed the trial had a significantly lower IQ, lower frequency of depression, and higher frequency of psychosis that could have moderated the outcome (eTable 1 in Supplement 2). Additionally, given the small sample size, the study was unable to control for psychiatric comorbidities and concomitant medications known to affect glutamate levels. Furthermore, predictive modeling could not be performed due to the limited sample size. Given the robustness of these findings, future studies would benefit from recruiting larger samples to enable additional analyses, such as predictive modeling with cross-validation based on pgACC glutamate levels. Finally, an independent sample would be beneficial to assess predictive accuracy.

## Conclusions

This short-term, parallel-design, double-blind RCT demonstrated that acute treatment with memantine in youths with ASD without intellectual disability was well tolerated and was associated with significant improvement in autistic behaviors. Importantly, higher pgACC glutamate levels were associated with a greater rate and magnitude of response to memantine, providing high efficiency at identifying treatment response. Although these results are promising, definitive conclusions regarding the efficiency of pgACC glutamate levels as a therapeutic biomarker await larger controlled trials that prospectively examine memantine response in individuals with autism based on the levels of pgACC glutamate concentration.

## Notes

Supplement 1.
Trial Protocol

Supplement 2.
eMethods.

eResults.

eReferences.
**eTable 1.** Demographics, Neurocognitive, Clinical, and Treatment Characteristics as Compared Between Participants With Complete and Incomplete Trial Data **eTable 2.** Treatment Efficacy Response **eTable 3.** Treatment Response Based on Pregenual Anterior Cingulate Cortex (pgACC) Glutamate Levels **eFigure.** AUC Response to Treatment Based on the Baseline Pregenual Anterior Cingulate Cortex (pgACC) Glutamate Levels

Supplement 3.
Data Sharing Statement
