# Supplementary material for: Memantine to Treat Social Impairment in Youths With Autism Spectrum Disorder: A Randomized Clinical Trial
Source: JAMA Netw Open. 2025 Oct 1;8(10):e2534927. doi: 10.1001/jamanetworkopen.2025.34927 (PMC12489667; doi:10.1001/jamanetworkopen.2025.34927)
Supplement: Supplement 1. — Trial Protocol [file jamanetwopen-e2534927-s001.pdf]

Study Protocol: Behavioral and Neural Response to Memantine in Adolescents with Autism Spectrum Disorders

Principal Investigator: Gagan Joshi, MD
Massachusetts General Hospital
Clinical and Research Program in Pediatric Psychopharmacology

Version Date: Initial Submission RtR, submitted to the IRB on 11/6/2013
Last Modified: 11/6/2013

I. BACKGROUND AND SIGNIFICANCE

ASDs are a group of neurodevelopmental disorders characterized by difficulties with socialization & reciprocal communication, along with restricted, repetitive behavior[1]. An increasingly higher prevalence of ASDs is documented in each successive epidemiological survey and is now estimated to affect more than 1% of youth[2].

Social Deficits in Autism: Deficits in social interaction are the central feature of autism and often result in significant impairment in cognitively capable individuals with ASDs. In light of the growing recognition of ASDs in intellectually capable individuals of all ages, there is an acute need for effective treatment for social deficits. Although there are drugs proven to be effective in treating target symptoms of hyperactivity, irritability, & repetitive behaviors in ASDs, to date, no medications have consistently been shown to reliably improve social impairments in ASDs[3-8]. Earlier studies of fenfluramine, secretin, & naltrexone have largely been disappointing[9-12]. Emerging evidence on the safety & efficacy of glutamatergic agents for the treatment of social deficits in ASDs is encouraging.

Glutamatergic Dysregulation in Autism: Glutamate (Glu) is the primary excitatory amino acid neurotransmitter in the brain. Glu, through its activity at N-methyl-D-aspartate (NMDA) receptors, is crucial for neurodevelopmental processes, including neuronal plasticity & higher cognitive functioning[13]. Over-activation of Glu is associated with excitotoxicity & apoptosis. Dysregulation in glutamatergic activity has been hypothesized to contribute to the pathophysiology of ASDs[14-15]. Evidence for increased Glu activity in autism comes from serological, postmortem brain, and preliminary genetic studies[16-24]. ^{1}HMRS in autism provides *in vivo* evidence of abnormal glutamatergic brain activity in autism by quantifying Glu levels in combination with glutamine (Gln) & gamma-aminobutyric acid (GABA; Glu+Gln+GABA=Glx). Although no Glx abnormality was identified in the temporal lobes or anterior cingulate cortex (ACC) by 1.5 T ^{1}HMRS in preschool-age children with ASDs lower levels of Glx were observed in grey matter by 3T ^{1}HMRS in older school-age children with ASDs[25-27]. On the contrary, in adults with ASDs, Glx levels are found to be higher in the amygdalo-hippocampal region (on 1.5 T ^{1}HMRS) and decreased in the right ACC (on 3T ^{1}HMRS)[28-29]. More recently, Harada & colleagues[30] specifically examined Glu metabolites at 3T ^{1}HMRS in children with ASDs and noted no abnormality in Glu levels in the frontal lobe & lenticular nuclei. Taken together, previous ^{1}HMRS studies in autism suggest Glx dysregulation in various regions, including regions implicated in autism (i.e., medial temporal lobe [MTL] & ACC). Our ^{1}HMRS study in adolescent males with HF-ASDs suggest significantly increased Glu in the ACC with no change in the bilateral MTL regions[31].

Role of Glutamate Modulators in the Treatment of Autism: Glutamatergic agents lamotrigine, amantadine, & D-cycloserine have been studied as potential treatments for symptoms of ASDs. Lamotrigine attenuates Glu release by inhibiting voltage-sensitive sodium channels in the presynaptic neuronal membranes[32]. In a randomized-controlled trial (RCT) of lamotrigine in 28 children with ASDs, although there was marked improvement in autism behaviors, an equally robust response to placebo (PBO) resulted in the lack of a statistically significant separation between the two groups[33]. Amantadine,

an antagonist at NMDA receptors, has also been studied in ASDs. In a RCT of amantadine in 39 youth with ASDs, amantadine was well tolerated and although amantadine was not superior to PBO in treating hyperactivity & irritability per parent report, it was associated with significant improvement in inappropriate speech & illness severity on clinician-rated measures of response[34]. D-cycloserine is a partial agonist at NMDA receptors[35]. In a single-blind PBO lead-in trial D-cycloserine was well tolerated & was associated with significant improvement in social withdrawal[36]. Thus, the empirical evidence for the efficacy of these glutamatergic agents for the treatment of social deficits in autism is modest at best. Memantine is a glutamatergic agent with a unique mechanism of action & the preliminary findings on safety & effectiveness are encouraging[37-38].

Memantine: Memantine Hydrochloride is a moderate-affinity, non-competitive, NMDA receptor antagonist. Memantine treatment in adults with Alzheimer's disease improves cognition, as well as functional & behavioral symptoms[39-40]. Available data from limited retrospective & prospective treatment studies of memantine in individuals with ASDs report an acceptable tolerability profile with improvement in a range of behavioral impairments including attention, hyperactivity, language, eye contact, social interaction & withdrawal, & repetitive behaviors[37-38].

Brain regions implicated in Autism: Several studies aiming to identify the etiology of autism have indicated involvement of limbic system structures including the amygdala, hippocampus, & the ACC[41-43]. These limbic structures also show a high affinity for NMDA receptor binding. ACC: is functionally associated with information processing and response to emotional cues and is, therefore, a region of interest in autism. The ACC also has close anatomic connections to the amygdala & participates in emotional regulation. Lesions of ACC are known to cause blunted affect, disinhibition, disabling repetitive behaviors, & impaired social judgment including the inability to interpret social cues[44]. Converging evidence from various investigative modalities suggests abnormalities in the ACC region in individuals with ASDs including histopathological changes of increased cell packing density & decreased cell size, smaller in volume, decrease in regional cerebral blood flow, & metabolically less active[34-35, 38, 39-40] with abnormal functional activity (theory of mind task related & resting state)[45-52]. MTL: structures, the hippocampus & amygdala, play crucial roles in associative memory & social cognition, respectively, & lesions in the MTL are implicated in social impairments intrinsic to ASDs[53-55]. Converging evidence of abnormalities in the MTL in ASDs comes from various histopathological & imaging studies revealing decreased neuronal size and increased cell density and cytoarchitectural minicolumnar pathology, bilateral decreased volume & hypo-perfusion, and abnormal activation especially of amygdala on face recognition[41,45-46,48, 55-65].

II. SPECIFIC AIMS

Primary Aims

Aim 1) To examine the clinical efficacy & tolerability of memantine monotherapy for the treatment of social impairment in adolescents with ASDs. We will study the short- & long-

term clinical effects of memantine in 40 adolescents with ASDs by conducting a 12-week randomized-controlled trial (RCT).

Aim 2) To examine the effect of memantine therapy on neural function in adolescents with ASDs. We will assess neural response to memantine therapy by measuring spectroscopic & resting state functional connectivity (RsFc) changes with memantine treatment. We will also assess association of neural & clinical response to memantine therapy.

Aim 3) To characterize neural functional deficits and/or abnormalities in adolescents with ASDs by comparing ASD subjects to healthy control subjects undergoing the same spectroscopic & RsFc imaging with two scans 12 weeks apart.

III. LENGTH OF STUDY

This study may take up to 16 weeks from enrollment (allowing up to four weeks to schedule and complete the initial screening process). Once subjects have completed the screening process and baseline characterization, they will complete a baseline scan (for ASD subjects, this scan will be pre-treatment). ASD subjects will then begin the 12-week randomized-controlled trial. All subjects, including healthy controls, will receive a follow-up scan approximately 12 weeks after the baseline scan. For ASD subjects, this scan will take place within one week of the final study visit (Week 12/early termination). Memantine responders and placebo non-responders who complete all 12 weeks of the RCT will be offered enrollment in a separate 12-week open-label extension protocol.

IV. SUBJECT SELECTION CRITERIA

A. Inclusion Criteria (all participants)

1. Male & female ages 13-17 yrs.
2. Tanner stage of ≥III (by Petersen Pubertal Development Scale[66]).

<u>Participants with ASDs</u>

3. DSM-IV-TR PDD diagnostic criteria for autistic disorder, Asperger's disorder, or PDD-NOS as established by clinical diagnostic interview
4. At least moderate severity of social impairment as measured by a score of ≥14 on the caregiver (parent/guardian)-completed **Social Withdrawal subscale of the Aberrant Behavior Checklist**[69] (ABC-SW) and a score of ≥4 on the clinician-administered **Clinical Global Impression-Severity scale (CGI-S)**.
5. Psychotropic medication-free for ≥4 weeks prior to trial participation.

<u>Healthy Control Participants</u>

3. Age-, sex-, & IQ-matched.
4. No Axis I diagnoses as established by the **Kiddie Schedule for Affective Disorders and Schizophrenia—Epidemiolgoical Version (K-SADS-E)**[70] & confirmed by clinical diagnostic interview.

5. No significant traits of ASDs as screened by **Social Responsiveness Scale-Second Edition (SRS-2)**[71] (raw score <60).

Exclusion Criteria (all participants)

1. IQ <85
2. Impaired communicative speech
3. Subjects currently treated with medication with primary central nervous system activity that are unwilling to withhold medication for 48 hours prior to scan.
4. Contraindications to MR scanning (claustrophobia, braces, metal in the body, etc.)
5. Subjects who are pregnant and/or lactating.
6. Subjects with a history of or a current seizure disorder.
7. Subjcets with a history of or a current liver or kidney disease.

V. SOURCE OF SUBJECTS

We propose to enroll up to 60 subjects with ASDs and 30 healthy controls for a total of 90 subjects. Up to 60 psychotropic drug-free adolescents with ASDs who exhibit marked social impairment will be enrolled in order to randomize 40 eligible subjects. In addition, up to 30 adolescents with no history of major psychiatric disorders, including ASDs, will enroll in order for 20 eligible age-, sex-, & IQ-matched subjects to participate as neuroimaging healthy controls. Subjects will be recruited from the referral pool of existing and new patients at three different MGH sites: the Bressler Program for ASDs, the Lurie Center for Autism, & the child psychiatry outpatient clinic.

VI. SUBJECT ENROLLMENT

Informed consent/assent will be obtained prior to the performance of any protocol procedures and prior to administration of study drug for ASD subjects. The informed consent and assent documents will be used to explain, in simple terms, the risks and benefits of study participation to the subject and their parent/guardian. The nature of the study will be fully explained to the subject and his/her parent/guardian by a board-certified physician who is either the principal investigator or a co-investigator. The subject and his/her parent/guardian will be encouraged to ask questions pertaining to their participation in the study and the subject and his/her parent/guardian may take as much time as they feel necessary to consider their participation in the study, as well as to consult with family members or their physician. Participation in this study is voluntary and the subject and/or his/her caretaker may withdraw the subject from the study at any time. The IRB-approved informed consent/assent documents will be signed and dated by the subject's parent/guardian, the subject, and the physician obtaining consent.

VII. STUDY PROCEDURES

This study includes two components: a 12-week randomized-controlled trial of memantine monotherapy for the treatment of Autism Spectrum Disorders, and two ^{1}HMRS scanning sessions pre- and post-treatment.

After providing written informed consent and assent, all subjects will complete a clinical diagnostic interview with a study clinician to assess eligibility. All subjects will be administered a detailed assessment battery including an indirect structured diagnostic interview (K-SADS-E), the SRS, and the Stanford-Binet Intelligence Scales to assess cognitive capabilities.

All participants will be required to give a urine sample to test for certain types of drugs. This includes prescription drugs, illegal drugs (street drugs), and controlled substances (substances that may be habit forming) that may affect behavior and that may be regulated by law. Results of the drug screen will be conveyed to the participant by the study clinician and if the results are positive for drug(s) there will be further discussion with the participant to determine if they are appropriate to participate in the trial.

In addition, female ASD and control subjects of childbearing potential will have a urine pregnancy test. If a participant has a positive urine pregnancy test, she will not be able to take part in the study. The study doctor will inform the participant of any positive test results. The decision whether to inform the subject's parent/guardian of these results will be made by the study doctor based on the participant's age and maturity level.

ASD subjects will also be assessed with the Austim Diagnostic Interview-Revised (ADI-R)[67], the Autism Diagnostic Observation Schedule (ADOS)[68], and the Clinical Evaluation of Language Fundamentals-Fourth Edition (CELF-4). ASD subjects will complete physical assessment measures (complete physical examination, vitals, height, ECG, and blood screening tests). For a complete schedule of assessments, refer to the Table I (page 18).

12-Week RCT (ASD Subjects only):

Participating adolescents with ASDs who meet the eligibility criteria will be randomly assigned to either memantine or placebo for the course of the 12-week RCT. Subjects will be assessed weekly during the titration phase (Weeks 1-4) & during the maintenance phase at midpoint (Week 6), Week 9, & at completion (Week 12/early termination). The same caretaker (a parent/guardian most familiar with the subject's day-to-day behavior) will participate in the assessment of behavioral symptoms at all visits of the treatment phases. In order to maintain the double-blind status of the trial, efficacy measures will be administered by a rating psychiatrist who is blind to the information on the tolerability, safety, & study medication titration.

Randomization: ASD subjects will be randomized to either active memantine or placebo in a 1:1 ratio after they have been determined to meet all eligibility criteria. Randomization lists will be generated by the statistician for each gender separately & passed to the investigational pharmacy for assignment.

Washout Period: ASD subjects who are currently being treated with any psychotropic medications must discontinue the use of their medication to be eligible for participation in this study. Medication washout is recommended by our clinicians to participants, their parent/guardian, and current providers – this is done based on a case-by-case assessment, considering the duration on drug, the dose, and the adverse effects associated with the treatment and effects of stopping that medication/treatment. Our office does not take over care for the patient, but remains available during this time period. The washout schedule –which will span four weeks or more -- will be discussed with the participant, their parent/guardian, and current providers. Individuals taking a medication that is effectively and safely treating their symptoms will not be taken off of such medication for the purpose of enrolling in this study.

Trial Phase (Weeks 0-12): Participants will be prescribed study medication per randomization for the period of 12 weeks. At each visit, safety & efficacy will be assessed by administering measures of efficacy (ABC, CGI & DSM Global Assessment of Functioning Scale [GAF]), tolerability (assessing treatment-emergent AEs), & safety (vital signs [blood pressure, pulse, weight]). At baseline & endpoint (completion/drop-visit), the following assessment measures will be administered: SRS-2, Children's Yale-Brown Obsessive Compulsive Scale modified for PDD (CY-BOCS-PDD), ADHD-Rating Scale (ADHD-RS), Children's Depression Rating Scale-Revised (CDRS-R), Child & Adolescent Symptom Inventory-4R-Anxiety (CASI-Anx), Pediatric Quality of Life Inventory (PedsQL), Diagnostic Analysis of Nonverbal Accuracy (DANVA 2), & Vineland Adaptive Behavior Scale (VABS). In addition, SRS & CY-BOCS-PDD will also be administered at midpoint. At the endpoint visit of the trial, participants will be reevaluated on the physical assessment measures.

Dose Titration Phase (Weeks 1-4): Study medication will be initiated at 2.5 mg/day, will be raised to 5 mg/day on day 4, and will be gradually up-titrated by 5 mg/wk to a maximum dose of 20 mg/day. Titration of the study medication is flexible, guided by clinical assessment of tolerability with the option for slower, lower, or hold titration. Memantine will be administered in divided dosages with a total daily dose of $\geq$2.5 mg/day.

Study Medication Dosing: Study medication (memantine/placebo) will be titrated to the maximum daily dose during the first 4 weeks of the trial (dose titration phase). Week 4 onwards, subjects will be maintained on maximum achieved dose until the end of the trial (dose maintenance phase; Weeks 5-12/early termination).

Memantine Flexible Titration Schedule

Visit	Day	Maximum Total Dose Prescribed (mg/day)
0	1	2.5
	5	5
1	8	10
2	15	15

3	22	20
4	28	Maintained on maximum achieved dose

Primary Outcome Measure of Efficacy: Clinician-rated CGI-Improvement (CGI-I) subscale & the parent-rated ABC-SW subscale. Treatment responders will be defined as improvement of ≥25% on the ABC-SW subscale <u>*and*</u> a score of 2 or 1 on the CGI-I subscale, i.e., “much” or “very much improved.”

Secondary Outcome Measures of Efficacy: As with other psychotropic treatment outcomes in this population[72], response to memantine may not be uniform for all domains of ASDs. The response differential will be examined by assessing change in the severity of the subscales of the SRS representing core domains of ASDs. The CY-BOCS-PDD[73-74] will be administered to assess change with treatment in the ritualistic behaviors associated with ASDs. Change with treatment in the level of adaptive functioning will be assessed by administering the VABS[75] to the caregiver. Change in the level of global functioning will be assessed by the clinician-rated GAF[76]. Change with treatment in the quality of life will be assessed by the caregiver-rated PedsQL[77]. Considering that features of ADHD, anxiety, & depression are frequently associated with ASDs[4] & there is evidence of memantine’s effectiveness in treating ADHD in adults, these symptoms will be assessed by administering the clinician-rated ADHD-RS[78], CDRS-R[79], & CASI-Anx[80].

Safety & Tolerability Outcome Measures: The safety & tolerability of memantine monotherapy will be monitored by administering a complete physical examination, battery of blood tests, urine tests & ECG at baseline & endpoint of the trial, and by recording AEs and obtaining vital signs at each study visit.

Drop Criteria: A subject may be withdrawn from the study at any time if any of the following conditions are met:

- Worsening of PDD, ADHD, anxiety, depression, mania, OCD or psychosis, as reflected by respective Clinical Global Improvement score of 6 (Much worse) or 7 (Very Much Worse) for 2 visits in a row.
- Subjects who experience intolerable adverse effects, and/or clinically significant laboratory values inconsistent with continuation in the study as determined by PI.
- Unstable psychiatric condition that clinically requires 1) treatment with prohibited concomitant psychotropic medications or 2) subjects requiring inpatient psychiatric admission.
- Emergent suicidality
- Active substance abuse
- Pregnancy
- Allergic drug reaction

- Non-compliance (less than 70% compliance for 2 visits or longer based on parent report)
- Failure to return medication for 2 consecutive visits
- Failure to keep study appointments for more than 2 consecutive visits without justification
- Clinical judgment of the investigator
- Withdrawal of consent

If study participation is discontinued due to safety reasons, participants will receive three follow-up visits, giving adequate time for appropriate psychiatric referrals to treaters in their community. Subjects who discontinue due to non-compliance with the protocol will receive a referral to ASD treaters in the area.

^{1}HMRS Scanning (ASD and Healthy Control Subjects):

All subjects will complete two scanning sessions at the McLean Imaging Center over the course of the study. For subjects with ASDs, the initial scan will take place after evaluation and screening and prior to randomization. Within one week of completing the 12-week RCT (or at study termination, if subjects do not complete all 12 weeks), ASD participants will have a second scanning session at McLean.

Upon signing consent/assent at MGH, healthy control subjects will participate in screening procedures to assess eligibility. Those who meet eligibility criteria and complete screening procedures will participate in two scanning sessions at McLean Imaging Center. The first scanning session will take place after screening procedures are completed at MGH, and the second scanning session will take place approximately 12 weeks after the first.

Additional IRB-approved consent and assent documents will be reviewed, signed and dated by all subjects, their parents/guardians, and a qualified member of study staff at McLean Imaging Center, where the scans will be performed. At this time, the additional informed consent and assent documents will be used to explain in simple terms the risks and benefits of the scanning procedures to the subject and their parent/guardian. The subject and their parent/guardian will be encouraged to ask questions and may take as much time as they feel necessary to consider their participation. Participation in this study is voluntary and the subject and/or his/her parent/guardian may withdraw the subject from the study at any time. The IRB-approved informed consent/assent documents will be signed and dated by the subject's parent/guardian, the subject, and the qualified McLean staff member obtaining consent.

ASSESSMENTS (see Table I)

Autism Diagnostic Observation Schedule (ADOS)[67]

- The ADOS is a semi-structured assessment of communication, social interaction, and play (or imaginative use of materials) for individuals suspected of having autism or other pervasive developmental disorders.

- Consists of four modules, each of which is appropriate for children and adults of differing developmental and language levels, ranging from nonverbal to verbally fluent.

Autism Diagnostic Interview-Revised (ADI-R)[68]
- The ADI-R is a structured interview conducted with parents/guardians of individuals suspected of having autism or other pervasive developmental disorders. The interview is used for diagnosing autism, planning treatment, and distinguishing autism from other developmental disorders.
- Consists of 93 questions that address reciprocal social interaction, communication, and language and restricted and repetitive interests and behaviors.

Kiddie Schedule for Affective Disorders and Schizophrenia-Epidemiologic Version (K-SADS-E)[70]
- This is a widely used, semi structured, diagnostic interview with established psychometric properties. It can be effectively administered by clinicians and/or trained non-clinician interviewers in 45 to 90 minutes, although more complex cases may require additional time.
- For all subjects, psychiatric data will be collected from the mother, or primary caretaker. All children and primary caregivers will be seen in direct clinical interview with the treating clinician.
- The diagnostic structured interview data will be collected using DatStat IllumeTM, a platform for electronic data capture that streamlines data collection and management, and ensures data integrity, resulting in improved data quality. The DatStat software allows researchers to design and implement study surveys for collecting, storing, retrieving, and manipulating data electronically. Research staff will enter interview responses into electronic assessment forms, and the responses are then transmitted securely via encrypted connection and stored in a secured database. This electronic data capture obviates the need for subsequent data entry by staff, thus minimizing human error.
- These interviews are completed securely via the internet by using any device with standard web access and browsers.

Clinical Evaluation of Language Fundamentals-Fourth Edition (CELF-4)[81]
- The CELF-4 is an individually administered test for determining if an individual (5-21 years old) has a language disorder or delay.
- Assesses four aspects of language (morphology and syntax, semantics, pragmatics, and phonological awareness) and can be administered in 30-60 minutes.

Stanford-Binet Intelligence Scales-Fifth Edition (SB5)[82]
- SB5 is an individually administered assessment of intelligence and cognitive

abilities for individuals ages 2-85+ years
- Assesses Fluid Reasoning, Knowledge, Quantative Reasoning, Visual-Spatial Processing, and Working Memory.

Cambridge Neuropsychological Test Automated Battery (CANTAB)[83]

CANTAB is designed to assess executive functioning. The subtests we will use are not sensitive to practice effects. Subtests will include the following:
- Working Memory and Planning
  - Spatial Working Memory (SWM): tests comprehension, learning, and reversal.
  - Stockings of Cambridge (SOC): assesses spatial planning and motor control.
- Attention
  - Intra-Extra Dimensional Set Shifting (IED): tests rule acquisition and attentional set shifting.
  - Reaction Time (RTI): measures speed of response.
- Rapid Visual Information Processing (RVP): tests sustained visual attention.
- Affective Go/No-go (AGN): assesses information processing biases for positive and negative stimuli.
- Verbal Recognition Memory (VRM): assesses immediate free recall, and immediate and delayed recognition memory.

Diagnostic Analysis of Nonverbal Accuracy Scale (DANVA 2)[84-85]

Consists of 4 tasks of social competence that includes tests for recognizing feelings expressed through faces and paralanguage by adults and children: 1) Child Faces 2) Child Paralanguage 3) Adult Faces 4) Adult Paralanguage. Child and Adult Facial Expressions subtests are used to assess face-emotion labeling. Each computer-administered subtest includes 24 photographs of child or adult models (12 female, 12 male per subtest) displaying equal numbers of high- and low-intensity expressions of happiness, sadness, anger, and fear. Faces appear for 2 seconds. In a forced-choice format, participants indicate by button-press which emotion a face expresses. Both subtests have been standardized and have acceptable internal consistency and reliability[82-83]. This test can be administered for testing social competence in children as young as 3 years of age.

Rating Scales

Parent Rated:
- Social Responsiveness Scale-Second Edition (SRS-2)[71] a 65-item rating scale completed by the parent used to measure the severity of autism spectrum symptoms as they occur in natural settings.
- Behavior Rating Inventory of Executive Function (BRIEF-Parent)[86] a 78-item rating scale to assess level of executive function deficits.

- Pediatric Quality of Life Inventory (PedsQL)[77] a 16-item caregiver-rated questionnaire to evaluate a participant's quality of life
- Aberrant Behavior Checklist[69] a 58-item scale completed by the parent and reviewed by a clinician to establish the frequency of problematic or abnormal behaviors
- Vineland Adaptive Behavior Scales-Second Edition (VABS)[75] a caregiver-rated scale used to measure a participant's level of adaptive and global psychosocial functioning

Clinician Rated:

Clinical Global Impression Scale (CGI)[87]
- The CGI is a measure of illness severity, improvement, and efficacy of treatment (National Institute of Mental Health, 1985). The score for severity ranges from 1 (normal, not at all ill) to 7 (among the most extremely ill subjects). Improvement ranges from 1 (very much improved) to 7 (very much worse). And lastly, the effectiveness index measures to what extent the subject is experiencing therapeutic effects as well as the level of adverse events they are experiencing. The CGI scale will be used for the assessment of global functioning. Additionally, the following disorder-specific CGIs will also be administered: PDD, ADHD, OCD, psychosis, mania, depression, and anxiety.

Depression
- The Children's Depression Rating Scale (CDRS)[79] is a widely used observational rating measure of depression severity in children and adolescents.
- CGI-Depression.

Anxiety
- Children and Adolescenty Symptom Inventory-4R Anxiety Scale (CASI-Anx)[80]
- CGI-Anxiety.

ADHD
- ADHD Symptom Checklist[78] assesses each of the individual symptoms of ADHD in DSM-IV (0-3 on a scale of severity)
- CGI-ADHD.

Psychosis
- CGI-Psychosis.

Obsessive-Compulsive Disorder (OCD)

- The Children's Yale Brown Obsessive Compulsive Scale for PDD (CY-BOCS-PDD)[74] will be used to assess obsessive and compulsive symptoms. This is a clinician-rated 10-item scale (total range from 0 to 40), with subtotals for obsessions (items 1-5) and compulsions (items 6-10).
- CGI-OCD.

Pervasive Developmental Disorder (PDD)

- CGI-PDD.
- CGI-PDD – Social Interaction.
- CGI-PDD – Mannerisms.
- CGI-PDD – Social Communication.
- MGH ASD Symptom Checklist (MGH-ASD-SCL): The spectrum of ASD symptoms will be screened by using clinician administered MGH-ASD-SCL. This screening instrument adopted items from DSM-IV diagnostic criteria for ASD and assesses for the individual core domains and associated features of ASD.
- MGH ASD Rating Scale (MGH-ASD-RS): Severity of ASD will be assessed by administering the clinician rated MGH-ASD-RS. This is a 19-item scale that assesses for the severity of core and associated symptoms of ASD in a likert scale from 0 to 3.

Level of Functioning

- DSM-IV Global Assessment of Functioning Scale (GAF)[76]: a composite rating of an individual's overall level of functioning (1= worst to 100 = best).

Safety

- Adverse Experiences: to record any adverse health events experienced during the study, along with duration, severity, cause, treatment, and outcome.
- Concomitant Medications: to record additional medications taken during the study.

VI. BIOSTATISTICAL ANALYSIS

Clinical Data Analysis

Because this is a RCT following subjects over a short period of time, missing data are not expected to impact our analyses such that standard statistical tests will be employed. Changes in the primary outcome measures of efficacy (CGI-I & ABC-SW subscale) within & between study groups over time will be tested with longitudinal generalized estimating equation (GEE) regression models (RM) estimated using STATA 12.0 within the framework of the general linear model (GLM). For binary outcomes, logistic RMs will be fit with the binomial family & the logit link. For count data, Poisson RMs will be fit with the Poission family & the log link, for normally distributed data, linear RMs will be fit with the Gaussian distribution & identity link. Each model will predict outcome scores from

treatment group (memantine vs. PBO), study visit (ordinal predictor), & the group by visit interaction, which is our test of efficacy. Secondary outcome measures (SRS, GAF, VABS, ADHD-RS, CY-BOCS-PDD, CDRS-R, CASI-Anx, & PedsQL) will be examined using models similar to the primary outcome measures. Changes in the tolerability outcome measurements will be tested using Chi-squared tests. All analyses will be intention to treat (ITT). For the extension phase, as in the acute phase, longitudinal GEE RMs will be used.

VII. RISKS AND DISCOMFORTS

Risks of Taking Memantine:

The most frequently observed adverse reactions with memantine are dizziness, confusion, headache, constipation, hypertension, cough, pain, hallucinations, somnolence, vomiting, dyspnea, and fatigue. Serious adverse reactions include Stevens-Johnson syndrome and seizures. All participants will be closely monitored for serious adverse reactions and drug-drug interactions with their ongoing concomitant medications. All serious unexpected adverse experiences of a research subject will be reported to the Human Studies Office at MGH.

Problems and side effects not listed above and not known at this time could occur. Subjects will be told of any changes in the way the study will be done and any newly discovered risks to which they may be exposed.

Risks of Blood Draws:

When blood is drawn, some discomfort may be associated with it at the time of the blood draw. Bruising and/or bleeding at the needle site may occur. Occasionally a person feels faint. Rarely, an infection may develop. If an infection does occur, it can be treated. A topical anesthetic cream (Topicaine, EMLA, or ELA-MAX) will be applied to numb the skin where blood will be drawn if subjects prefer.

Risks of ^{1}HMRS:

^{1}HMRS is not associated with any known adverse effects except to people with metal or magnetic implants (such as metal clips from surgery or a cardiac pacemaker). Therefore, if a subject has such metal objects in her/his body, s/he will be excluded from this study.

There are no known risks of scanning for fetuses. However, the safety of scans for pregnant women and nursing mothers has not been established. Therefore, subjects must have a negative pregnancy test prior to each scan and nursing mothers cannot participate. If a participant has a positive pregnancy test, the study doctor will inform

the subject and she will not be able to take part in the study. The decision whether to inform the parent of these results will be made by the physician based on the participant's age and maturity level and the requirements of the law, unless the participant agrees to parental notification. Birth control will be required for subjects who are sexually active. There may be some risk of emotional distress in the event of a positive pregnancy test.

Risks of Assessments:

Some of the questions asked in this study may make subjects feel uncomfortable, and some of the neuropsychological testing may be boring or frustrating. While we hope subjects and their parents will answer all questions, they may skip any questions they do not wish to answer.

Adverse events and unanticipated problems will be reported to the PHRC according to current guidelines. We will follow and adhere to all guidelines as defined and outlined on the Partners Human Research Committee web site:

(http://healthcare.partners.org/phsirb/adverse_events.htm).

VIII. POTENTIAL BENEFITS

There may be no direct benefit to subjects participating in this study. Potential benefits to the participants include education about ASD, a trial of medication that could be continued after the study, and the opportunity to contribute to medical science and thus help others with the disorder.

All subjects, including healthy controls, may receive up to $150 for completing both scanning visits ($75 will be paid per completed scanning visit).

IX. REFERENCES

1. Association AP, DSM-IV Sourcebook. Vol. 1. 1994, Washington, DC: American Psychiatric Association.
2. Autism and Developmental Disabilities Monitoring Network Surveillance Year 2008 Principal Investigators; Centers for Disease Control and Prevention. Prevalence of autism spectrum disorders--Autism and Developmental Disabilities Monitoring Network, 14 sites, United States, 2008. MMWR Surveill Summ. 2012;61(3):1-19. PMID: 22456193.
3. Research Units on Pediatric Psychopharmacology Autism Network: Risperidone in children with autism and serious behavioral problems. N Engl J Med. 2002;347: 314-321. PMID: 12151468.
4. Research Units on Pediatric Psychopharmacology Autism Network. Randomized, controlled, crossover trial of methylphenidate in pervasive developmental disorders with hyperactivity. Arch Gen Psychiatry. 2005 Nov;62(11):1266-74. PMID: 16275814.

5. McDougle C, Holmes J, Carlson D, Pelton G, Cohen D, and Price L. A double-blind placebo-controlled study of risperidone in adults with autistic disorder and other pervasive development disorders. Arch Gen Psychiatry. 1998;55: 633-641. PMID: 9672054.
6. McDougle C, Naylor S, Cohen D, Volkmar F, Heninger G, and Price L. A Double-blind, Placebo-Controlled Study of Fluvoxamine in Adults with Autistic Disorder. Arch Gen Psychiatry. 1996;53: 1001-1008. PMID: 8911223.
7. Hollander E, Anagnostou E, Chaplin W, Esposito K, Haznedar MM, Licalzi E, Wasserman S, Soorya L, and Buchsbaum M. Striatal volume on magnetic resonance imaging and repetitive behaviors in autism. Biol Psychiatry. 2005;58: 226-32. PMID: 15939406.
8. Hollander E, Soorya L, Chaplin W, Anagnostou E, Taylor BP, Ferretti CJ, Wasserman S, Swanson E, and Settipani C. A double-blind placebo-controlled trial of fluoxetine for repetitive behaviors and global severity in adult autism spectrum disorders. Am J Psychiatry. 2012;169: 292-9. PMID: 22193531.
9. Campbell M, Adams P, Small AM, Curren EL, Overall JE, Anderson LT, Lynch N, Perry R. Efficacy and safety of fenfluramine in autistic children. J Am Acad Child Adolesc Psychiatry. 1988;27(4):434-9. PMID: 3053609.
10. Williams K, Wray JA, and Wheeler DM. Intravenous secretin for autism spectrum disorders (ASD). Cochrane Database Syst Rev. 2012;4: CD003495. PMID: 16034901.
11. Campbell M, Anderson L, Small A, Adams P, and Gonzalez N. Naltrexone in autistic children: Behavioral symptoms and attentional learning. J Am Acad Child Adolesc Psychiatry. 1993;32: 1283-1291. PMID: 8282676.
12. Willemsen-Swinkels S, Buitelaar JK, and van Engeland H. The effects of chronic naltrexone treatment in young autistic children: A double-blind placebo-controlled crossover study. Biol Psychiatry. 1996;39: 1023-1031. PMID: 8780837.
13. Cotman CW and Anderson AJ. A potential role for apoptosis in neurodegeneration and Alzheimer's disease. Mol Neurobiol. 1995;10: 19-45. PMID:7598831.
14. Carlsson ML. Hypothesis: is infantile autism a hypoglutamatergic disorder? Relevance of glutamate - serotonin interactions for pharmacotherapy. J Neural Transm. 1998;105: 525-35. PMID: 9720980.
15. McDougle CJ. Current and emerging therapeutics of autistic disorder and related pervasive developmental disorders. In: Davis KL, Charney D, Coyle JT, et al, eds. Psychopharmacology: The Fifth Generation of Progress. Philadelphia, PA: Lippincott Williams & Wilkins. 2002.
16. Shimmura C, Suda S, Tsuchiya KJ, Hashimoto K, Ohno K, Matsuzaki H, Iwata K, Matsumoto K, Wakuda T, Kameno Y, Suzuki K, Tsujii M, Nakamura K, Takei N, and Mori N. Alteration of plasma glutamate and glutamine levels in children with high-functioning autism. PLoS One. 2011;6: e25340. PMID: 3187770.
17. Lappalainen R and Riikonen RS. High levels of cerebrospinal fluid glutamate in Rett syndrome. Pediatr Neurol. 1996;15: 213-6. PMID: 8916158.
18. Blue ME, Naidu S, and Johnston MV. Altered development of glutamate and GABA receptors in the basal ganglia of girls with Rett syndrome. Exp Neurol. 1999;156: 345-52. PMID: 10328941.
19. Fatemi SH, Halt AR, Stary JM, Kanodia R, Schulz SC, and Realmuto GR. Glutamic acid decarboxylase 65 and 67 kDa proteins are reduced in autistic parietal and cerebellar cortices. Biol Psychiatry. 2002;52: 805-10. PMID: 12372652.
20. Jamain S, Betancur C, Quach H, Philippe A, Fellous M, Giros B, Gillberg C, Leboyer M, and Bourgeron T. Linkage and association of the glutamate receptor 6 gene with autism. Mol Psychiatry. 2002;7: 302-10. PMID: 2547854.
21. Shuang M, Liu J, Jia MX, Yang JZ, Wu SP, Gong XH, Ling YS, Ruan Y, Yang XL, and Zhang D. Family-based association study between autism and glutamate receptor 6 gene in Chinese Han trios. Am J Med Genet B Neuropsychiatr Genet. 2004;131: 48-50. PMID: 15389769.

22. Serajee FJ, Zhong H, Nabi R, and Huq AH. The metabotropic glutamate receptor 8 gene at 7q31: partial duplication and possible association with autism. J Med Genet. 2003;40: e42. PMID: 12676915.
23. Ramoz N, Reichert JG, Smith CJ, Silverman JM, Bespalova IN, Davis KL, and Buxbaum JD. Linkage and association of the mitochondrial aspartate/glutamate carrier SLC25A12 gene with autism. Am J Psychiatry. 2004;161: 662-9. PMID:15056512.
24. Purcell S and Sham P. Variance components models for gene-environment interaction in quantitative trait locus linkage analysis. Twin Research. 2002;5: 572-6. PMID: 12573188.
25. Friedman SD, Shaw DW, Artru AA, Richards TL, Gardner J, Dawson G, Posse S, and Dager SR. Regional brain chemical alterations in young children with autism spectrum disorder. Neurology. 2003;60: 100-7. PMID: 12525726.
26. Friedman SD, Shaw DW, Artru AA, Dawson G, Petropoulos H, and Dager SR. Gray and white matter brain chemistry in young children with autism. Arch Gen Psychiatry. 2006;63: 786-94. PMID: 16818868.
27. DeVito TJ, Drost DJ, Neufeld RW, Rajakumar N, Pavlosky W, Williamson P, and Nicolson R. Evidence for cortical dysfunction in autism: a proton magnetic resonance spectroscopic imaging study. Biol Psychiatry. 2007;61: 465-73. PMID: 17276747.
28. Page LA, Daly E, Schmitz N, Simmons A, Toal F, Deeley Q, Ambery F, McAlonan GM, Murphy KC, and Murphy DG. In vivo ^{1}H-magnetic resonance spectroscopy study of amygdala-hippocampal and parietal regions in autism. Am J Psychiatry. 2006;163: 2189-92. PMID: 17151175.
29. Bernardi S, Anagnostou E, Shen J, Kolevzon A, Buxbaum JD, Hollander E, Hof PR, Fan J. In vivo ^{1}H-magnetic resonance spectroscopy study of the attentional networks in autism. Brain Res. 2011;22: 198-205. PMID: 21185269.
30. Harada M, Taki MM, Nose A, Kubo H, Mori K, Nishitani H, and Matsuda T. Non-invasive evaluation of the GABAergic/glutamatergic system in autistic patients observed by MEGA-editing proton MR spectroscopy using a clinical 3 tesla instrument. J Autism Dev Disord. 2011;41: 447-54. PMID: 20652388.
31. Joshi G, Biederman J, Wozniak J, Goldin RL, Crowley D, Furtak S, Lukas SE, Gönenc A. Magnetic resonance spectroscopy study of the glutamatergic system in adolescent males with high-functioning autistic disorder: A pilot study at 4T. Eur Arch Psychiatry Clin Neurosci. 2012 Sep 18. [Epub ahead of print]. PMID: 22986449
32. Coulter DA. Antiepileptic drug cellular mechanisms of action: where does lamotrigine fit in? J Child Neurol. 1997;12 Suppl 1: 2-9. Review. PMID: 9429123.
33. Belsito KM, Law PA, Kirk KS, Landa RJ, and Zimmerman AW. Lamotrigine therapy for autistic disorder: a randomized, double-blind, placebo-controlled trial. J Autism Dev Disord. 2001;31: 175-81. PMID: 11450816.
34. King BH, Wright DM, Handen BL, Sikich L, Zimmerman AW, McMahon W, Cantwell E, Davanzo PA, Dourish CT, Dykens EM, Hooper SR, Jaselskis CA, Leventhal BL, Levitt J, Lord C, Lubetsky MJ, Myers SM, Ozonoff S, Shah BG, Snape M, Shernoff EW, Williamson K, and Cook EH, Jr. Double-blind, placebo-controlled study of amantadine hydrochloride in the treatment of children with autistic disorder. J Am Acad Child Adolesc Psychiatry. 2001;40: 658-65. PMID: 11392343.
35. D'Souza DC, Charney D, and Krystal J. Glycine Site Agonists of the NMDA Receptor: A Review. CNS Drug Rev. 1995;1: 227-260.
36. Posey DJ, Kem DL, Swiezy NB, Sweeten TL, Wiegand RE, and McDougle CJ. A pilot study of d-cycloserine in subjects with autistic disorder. Am J Psychiatry. 2004;161:2115-2117. PMID: 15514414.
37. Owley T, Salt J, Guter S, Grieve A, Walton L, Ayuyao N, Leventhal BL, and Cook EH. A prospective, open-Label trial of memantine in the treatment of cognitive, behavioral, and memory dysfunction in pervasive developmental disorders. J Child Adolesc Psychopharmacol. 2006;16: 517-24. PMID: 17069541.

38. Chez M, Hing P, Chin K, Memon S, and Kirschner S. Memantine experience in children and adolescents with autism spectrum disorders. Ann Neurol. 2004;56.
39. Reisberg B, Doody R, Stöffler A, Schmitt F, Ferris S, and Möbius HJ. Memantine in moderate-to-severe Alzheimer's disease. N Engl J Med. 2003;348:1333–41. PMID: 12672860.
40. Tariot PN, Farlow MR, Grossberg GT, Graham SM, McDonald S, and Gergel I. Memantine treatment in patients with moderate to severe Alzheimer disease already receiving donepezil: A randomized con-trolled trial. J Am Med Assn. 2004;291:317–24. PMID: 14734594.
41. Baron-Cohen S, Ring HA, Wheelwright S, Bullmore ET, Brammer MJ, Simmons A, Williams SC. Social intelligence in the normal and autistic brain: an fMRI study. Eur J Neurosci. 1999;11(6):1891-8. PMID: 10336657.
42. Bauman ML and Kemper TL. Neuroanatomic observations of the brain in autism: a review and future directions. Int J Dev Neurosci. 2005;23: 183-7. PMID: 15749244.
43. Sokol DK, Dunn DW, Edwards-Brown M, and Feinberg J. Hydrogen proton magnetic resonance spectroscopy in autism: preliminary evidence of elevated choline/creatine ratio. J Child Neurol. 2002;17: 245-9. PMID: 12088077.
44. Devinsky O, Morrell MJ, and Vogt BA. Contributions of anterior cingulate cortex to behaviour. Brain. 1995;118 (Pt 1): 279-306. PMID: 7895011.
45. Bauman M and Kemper TL. Histoanatomic observations of the brain in early infantile autism. Neurology. 1985;35: 866-74. PMID: 4000488.
46. Kemper TL and Bauman M. Neuropathology of infantile autism. J Neuropathol Exp Neurol. 1998;57: 645-52. PMID: 9690668.
47. Simms ML, Kemper TL, Timbie CM, Bauman ML, and Blatt GJ. The anterior cingulate cortex in autism: heterogeneity of qualitative and quantitative cytoarchitectonic features suggests possible subgroups. Acta Neuropathol. 2009;118: 673-84. PMID: 19590881.
48. Ohnishi T, Matsuda H, Hashimoto T, Kunihiro T, Nishikawa M, Uema T, and Sasaki M. Abnormal regional cerebral blood flow in childhood autism. Brain. 2000;123 (Pt 9): 1838-44. PMID: 10960047.
49. Haznedar MM, Buchsbaum MS, Metyger M, Solimando A, Spiegel-Cohen J, and Hollander E. Anterior cingulate gyrus volume and glucose metabolism in autistic disorder. Am J Psychiatry. 1997;154:8. PMID: 9247387.
50. Di Martino A, Ross K, Uddin LQ, Sklar AB, Castellanos FX, and Milham MP. Functional brain correlates of social and nonsocial processes in autism spectrum disorders: an activation likelihood estimation meta-analysis. Biol Psychiatry. 2009;65: 63-74. PMID: 18996505.
51. Silk TJ, Rinehart N, Bradshaw JL, Tonge B, Egan G, O'Boyle M W, and Cunnington R. Visuospatial processing and the function of prefrontal-parietal networks in autism spectrum disorders: a functional MRI study. Am J Psychiatry. 2006;163: 1440-3. PMID: 16877661.
52. Di Martino A, Scheres A, Margulies DS, Kelly AM, Uddin LQ, Shehzad Z, Biswal B, Walters JR, Castellanos FX, and Milham MP. Functional connectivity of human striatum: a resting state fMRI study. Cereb Cortex. 2008;18: 2735-47. PMID: 18400794.
53. Baron-Cohen S, Ring HA, Bullmore ET, Wheelwright S, Ashwin C, and Williams SC. The amygdala theory of autism. Neurosci Biobehav Rev. 2000;24: 355-64. PMID: 10781695.
54. Stone VE, Baron-Cohen S, Calder A, Keane J, and Young A. Acquired theory of mind impairments in individuals with bilateral amygdala lesions. Neuropsychologia. 2003;41: 209-20. PMID: 12459219.
55. Sweeten TL, Posey DJ, Shekhar A, and McDougle CJ. The amygdala and related structures in the pathophysiology of autism. Pharmacol Biochem Behav. 2002;71: 449-55. PMID: 11830179.
56. Raymond GV, Bauman ML, Kemper TL. Hippocampus in autism: a Golgi analysis. Acta Neuropathol. 1996;91(1):117-9. PMID: 8773156.

57. Casanova MF, Buxhoeveden DP, Switala AE, and Roy E. Minicolumnar pathology in autism. Neurology. 2002;58: 428-32. PMID: 11839843.
58. Bachevalier J and Loveland K, Early medial temporal dysfunction and autism, in Neurodevelopmental Mechanisms in Psychopathology, Cicchetti D and Walker EP, Editors. 2003, Cambridge University Press: Cambridge, England. 215-238.
59. Herbert MR, Ziegler DA, Deutsch CK, O'Brien LM, Lange N, Bakardjiev A, Hodgson J, Adrien KT, Steele S, Makris N, Kennedy D, Harris GJ, and Caviness VS, Jr. Dissociations of cerebral cortex, subcortical and cerebral white matter volumes in autistic boys. Brain. 2003;126: 1182-92. PMID: 12690057.
60. Aylward EH, Minshew NJ, Goldstein G, Honeycutt NA, Augustine AM, Yates KO, Barta PE, and Pearlson GD. MRI volumes of amygdala and hippocampus in non-mentally retarded autistic adolescents and adults. Neurology. 1999;53: 2145-50. PMID: 10599796.
61. Zilbovicius M, Boddaert N, Belin P, Poline JB, Remy P, Mangin JF, Thivard L, Barthelemy C, and Samson Y. Temporal lobe dysfunction in childhood autism: A PET study. Am J Psychiatry. 2000;157:1988-93. PMID: 11097965.
62. Boddaert N and Zilbovicius M. Functional neuroimaging and childhood autism. Pediatr Radiol. 2002;32: 1-7. PMID: 11819054.
63. Critchley HD, Daly EM, Bullmore ET, Williams SC, Van Amelsvoort T, Robertson DM, Rowe A, Phillips M, McAlonan G, Howlin P, and Murphy DG. The functional neuroanatomy of social behaviour: changes in cerebral blood flow when people with autistic disorder process facial expressions. Brain. 2000;123 (Pt 11):2203-12. PMID: 11050021.
64. Schultz RT, Gauthier I, Klin A, Fulbright RK, Anderson AW, Volkmar F, Skudlarski P, Lacadie C, Cohen DJ, and Gore JC. Abnormal ventral temporal cortical activity during face discrimination among individuals with autism and Asperger syndrome. Arch Gen Psychiatry. 2000;57: 331-40. PMID: 10768694.
65. Pierce K, Muller RA, Ambrose J, Allen G, and Courchesne E. Face processing occurs outside the fusiform 'face area' in autism: evidence from functional MRI. Brain. 2001;124: 2059-73. PMID: 11571222.
66. Petersen A, Crockett L, Richards M, and Boxer A. A self-report measure of pubertal status: Reliability, validity, and initial norms. J Youth and Adolesc. 1988;17: 117-133.
67. Lord C, Rutter M, and Le Couteur A. Autism Diagnostic Interview-Revised: a revised version of a diagnostic interview for caregivers of individuals with possible pervasive developmental disorders. J Autism Dev Disord. 1994;24: 659-85. PMID: 7814313.
68. Lord C, Rutter M, Goode S, Heemsbergen J, Jordan H, Mawhood L, and Schopler E. Autism diagnostic observation schedule: a standardized observation of communicative and social behavior. J Autism Dev Disord. 1989;19: 185-212. PMID: 11055457.
69. Aman MG, Singh NN, Stewart AW, and Field CJ. The aberrant behavior checklist: a behavior rating scale for the assessment of treatment effects. Am J Ment Def. 1985;89: 485-491. PMID: 3993694.
70. Orvaschel H. Schedule for Affective Disorder and Schizophrenia for School-Age Children Epidemiologic Version. 5th Edition ed. 1994, Ft. Lauderdale: Nova Southeastern University, Center for Psychological Studies.
71. Constantino JN & Gruber CP. The Social Responsiveness Scale-Second Edition. 2012, Los Angeles: Western Psychological Services.
72. McDougle CJ, Scahill L, Aman MG, McCracken JT, Tierney E, Davies M, Arnold LE, Posey DJ, Martin A, Ghuman JK, Shah B, Chuang SZ, Swiezy NB, Gonzalez NM, Hollway J, Koenig K, McGough JJ, Ritz L, and Vitiello B. Risperidone for the core symptom domains of autism: results from the study by the autism network of the research units on pediatric psychopharmacology. Am J Psychiatry. 2005;162: 1142-8. PMID: 15930063.
73. Goodman WK, Rasmussen SA, Price LH, Mazure C, Heninger GR, and Charney DS, Yale-Brown obsessive compulsive scale (Y-BOCS). 1986 (Rev 89), Yale University.

74. Scahill L, McDougle CJ, Williams SK, Dimitropoulos A, Aman MG, McCracken JT, Tierney E, Arnold LE, Cronin P, Grados M, Ghuman J, Koenig K, Lam KS, McGough J, Posey DJ, Ritz L, Swiezy NB, and Vitiello B. Children's Yale-Brown Obsessive Compulsive Scale modified for pervasive developmental disorders. J Am Acad Child Adolesc Psychiatry. 2006;45: 1114-23. PMID: 16926619.
75. Sparrow S, Balla D, and Cicchette D, Vineland Adaptive Behavior Scales. 1984, Circle Pines, MN: American Guidance Service Publishing.
76. Endicott J, Spitzer RL, Fleiss JL, and Cohen J. The global assessment scale. A procedure for measuring overall severity of psychiatric disturbance. Arch Gen Psychiatry. 1976;33(6):766-71. PMID: 938196.
77. Bastiaansen D, Koot HM, Bongers IL, Varni JW, and Verhulst FC. Measuring quality of life in children referred for psychiatric problems: psychometric properties of the PedsQL 4.0 generic core scales. Qual Life Res. 2004;13: 489-95. PMID: 15085921.
78. DuPaul G. The ADHD Rating Scale: Normative data, reliability, and validity. 1990. Unpublished manuscript, University of Massachusetts Medical Center, Worcester.
79. Poznanski E, Freeman L, and Mokros H. Children's depression rating scale-revised. Psychopharmacology Bulletin. 1985;21: 979-989.
80. Gadow KD and Sprafkin J. Adolescent symptom inventory-4 norms manual. 1998, Stony Brook, NY: Checkmate Plus.
81. Semel E, Wiig EH, & Secord WA. Clinical Evaluation of Language Fundamentals-Fourth Edition (CELF-4). 2008. Upper Saddle River, NJ: Pearson Education, Inc.
82. Roid GH & Barram RA. Essentials of Stanford-Binet Intelligence Scales (SB5) Assessment. 2004. Hoboken, NJ: John Wiley & Sons, Inc.
83. CANTABeclipse Test Administration Guide, 2004, Cambridge: Cambridge Cognition Limited. 1-164.
84. Nowicki, S., & Carton, J. (1993). The measurement of emotional intensity from facial expressions. Journal of Social Psychology, 133, 749–750.
85. Nowicki, S., & Duke, M. (1989). A measure of nonverbal social processing ability in children between the ages of 6 and 10. Paper presented at the American Psychological Society, Alexandria, VA.
86. Gioia GA, Isquith PK, Guy SC, Kenworthy L. Behavior Rating Inventory of Executive Function (BRIEF). 2000, Lutz, FL: Psychological Assessment Resources, Inc.
87. National Institute of Mental Health: CGI (Clinical Global Impression) Scale—NIMH. Psychopharmacol Bull 21:839–844, 1985.

Table I. Study Schema

**Week**	Screening	Pre-BL*	BL	1	2	3	4	6	9	12
**Consent**	**XX**									
**Procedures**										
Blood Draw	**X**									**X**
ECG	**X**									**X**
Physical Exam (including PPDS and waist circumference)	**X**									**X**
Height	**X**		**X**							**X**
Vital Signs (weight, BP, pulse)	**X**		**X**	**X**	**X**	**X**	**X**	**X**	**X**	**X**
Urine Drug Screen	**XX**							**X**		**X**
Urine Pregnancy (females only)	**XX**							**X**		**X**
**Characterization Assessments**										
ADOS	**X**									
ADI-R	**X**									
K-SADS-E	**XX**									
Clinical Interview	**XX**									
Stanford Binet	**XX**									
CELF-4	**X**									
DANVA 2			**X**					**X**		**X**
CANTAB			**X**					**X**		**X**
**Clinician Rated Scales**										
CGIs**	**X**		**X**	**X**	**X**	**X**	**X**	**X**	**X**	**X**
GAF			**X**	**X**	**X**	**X**	**X**	**X**	**X**	**X**
CY-BOCS-PDD			**X**					**X**		**X**
MGH-ASD-SCL	**X**									
MGH-ASD-RS			**X**					**X**		**X**
CDRS-R			**X**					**X**		**X**
ADHD-RS			**X**					**X**		**X**
CASI-Anx			**X**					**X**		**X**
Adverse Events		**XX**	**X**	**X**	**X**	**X**	**X**	**X**	**X**	**XX**
Concomitant Medications		**XX**	**X**	**X**	**X**	**X**	**X**	**X**	**X**	**XX**
**Patient/Parent-Rated Scales**										
VABS			**X**							**X**
ABC	**X**		**X**	**X**	**X**	**X**	**X**	**X**	**X**	**X**
BRIEF Parent			**X**					**X**		**X**
PedsQL			**X**							**X**
SRS	**XX**							**X**		**X**
**^{1}HMRS Scan**		**XX**								**XX**

X: ASD only tasks; XX: ASD and HC tasks; *BL: Baseline; **CGIs: General, PDD, PDD-SC, PDD-SI, PDD-M, ADHD, Anxiety, OCD, MDD, Mania, Psychosis.
