# Supplementary material for: Memantine to Treat Social Impairment in Youths With Autism Spectrum Disorder: A Randomized Clinical Trial
Source: JAMA Netw Open. 2025 Oct 1;8(10):e2534927. doi: 10.1001/jamanetworkopen.2025.34927 (PMC12489667; doi:10.1001/jamanetworkopen.2025.34927)
Supplement: Supplement 2. — eMethods. eResults. eReferences. eTable 1. Demographics, Neurocognitive, Clinical, and Treatment Characteristics as Compared Between Participants With Complete and Incomplete Trial Data eTable 2. Treatment Efficacy Response eTable 3. Treatment Response Based on Pregenual Anterior Cingulate Cortex (pgACC) Glutamate Levels eFigure. AUC Response to Treatment Based on the Baseline Pregenual Anterior Cingulate Cortex (pgACC) Glutamate Levels [file jamanetwopen-e2534927-s002.pdf]

Supplemental Online Content

Joshi G, Gönenc A, DiSalvo M, et al. Memantine to treat social impairment in youths with autism spectrum disorder: a randomized clinical trial. *JAMA Netw Open*. 2025;8(10):e2534927. doi:10.1001/jamanetworkopen.2025.34927

eMethods.

eResults.

eReferences.

eTable 1. Demographics, Neurocognitive, Clinical, and Treatment Characteristics as Compared Between Participants With Complete and Incomplete Trial Data

eTable 2. Treatment Efficacy Response

eTable 3. Treatment Response Based on Pregenual Anterior Cingulate Cortex (pgACC) Glutamate Levels

eFigure. AUC Response to Treatment Based on the Baseline Pregenual Anterior Cingulate Cortex (pgACC) Glutamate Levels

This supplemental material has been provided by the authors to give readers additional information about their work.

© 2025 Joshi G et al. *JAMA Network Open*.

eMethods.

Trial Design: Youth with autism spectrum disorder (ASD) who met the eligibility criteria were randomized to receive either memantine or placebo during the course of the 12-week trial. Participants underwent multimodal magnetic resonance imaging (MRI) scans prior to initiating the 12-week treatment trial. Participants were assessed weekly during the titration phase (baseline to Week 4) and during the maintenance phase at midpoint (Week 6), Week 9, and upon completion (Week 12). At each study visit, safety and efficacy were assessed by administering measures of efficacy (Clinical Global Impression [CGI], Global Assessment of Functioning scale), tolerability (treatment-emergent adverse events), and safety (vital signs, including weight). Compliance with study medication, concomitant medications, and psychosocial interventions was assessed at each study visit. At baseline, midpoint, and endpoint (completion/early termination) disorder specific severity assessment measures were administered for ASD (Social Responsiveness Scale-Second Edition School-Age Form [SRS-2], Social Withdrawal subscale of Aberrant Behavior Checklist, Children's Yale-Brown Obsessive Compulsive Scale modified for Pervasive Developmental Disorder) and frequently associated psychopathology (Attention Deficit Hyperactivity Disorder Rating Scale-IV, Children's Depression Rating Scale-Revised, Anxiety Subscale of the Child and Adolescent Symptom Inventory-5).
Eligibility Criteria for Participants: Intact intellectual ability was established by a full-scale intelligence quotient (IQ) of ≥85 based on the Vocabulary and Matrix Reasoning subtests of the Wechsler Abbreviated Scale of Intelligence-Second Edition (WASI-II).[1] To ensure inclusion of participants with intact intellectual functioning, this trial applied a minimum IQ threshold of greater than one standard deviation below the mean (i.e., IQ ≥85). This criterion was intended to include individuals with average or above-average intellectual functioning and to exclude those with borderline (IQ 70–84) or impaired (IQ <70) intellectual functioning. Concomitant treatment(s) with other psychotropic medications was allowed provided subjects had been receiving a stable dose for at least 4 weeks prior to initiating trial medication, and the dose remained stable throughout the trial. Psychosocial interventions were permitted to continue during the study, provided they were stable and ongoing for at least four weeks prior to the initiation of trial medication. Youth were excluded from participation who had a history of prior treatment with memantine or current treatment with glutamate-modulating agents (lamotrigine, amantadine, N-acetylcysteine, and D-cycloserine), or agents that affect memantine plasma levels (triamterene, metformin, hydrochlorothiazide, cimetidine, ranitidine, quinidine, nicotine).
Screening Assessments: Eligible subjects were administered a comprehensive assessment battery consisting of diagnostic, neuropsychological, and physical measures. In addition to the clinical diagnostic evaluation, a systematic assessment of ASD included administration of the Autism Diagnostic Interview-Revised[2] and the Autism Diagnostic Observation Schedule-Second Edition.[3] Associated psychopathology was assessed using the Kiddie-Schedule for Affective Disorders and Schizophrenia-Epidemiologic Version.[4] Full-scale intelligence quotient was assessed on the WASI-II. Physical measures included height, body weight, waist circumference, and Tanner staging on the Petersen Pubertal Development Scale[5] to establish the stage of sexual maturity. Physical assessment included a standard battery of blood tests, an electrocardiogram, a urine drug test, and a urine pregnancy test for women of child-bearing potential.
Study Intervention and Randomization: The Massachusetts General Hospital (MGH) Clinical Trials Pharmacy prepared the blinded memantine and placebo capsules for the study. Placebo capsules were compounded using lactose monohydrate NF powder as the filler and filled into empty size 0 blue capsules. To match the placebo in appearance, commercially available memantine tablets were micropulverized, mixed with lactose powder, and filled into size 0 blue capsules. Capsule weight checks were performed to ensure uniformity and dose accuracy. The MGH Clinical Trials Pharmacy dispensed the study medication (memantine and placebo) based on the randomization schedule. The randomization schedule remained confidential throughout the study. All participants and members of the study staff, including the clinicians and statistician, were blind to the intervention assignments throughout the duration of the study. Study medication was initiated at 2.5 mg/day, raised to 5 mg/day on day 4, and then up-titrated by 5 mg/week to a maximum dose of 20 mg/day. Except for the initial 2.5 mg once-daily starting dose, all subsequent doses were administered in divided daily dosages. Medication compliance was assessed via caretaker report at each study visit. A count of the returned study medication was verified against caretaker report and those who regularly missed doses were counseled to improve the medication compliance. In addition, rescue medications diphenhydramine (up to 50 mg) and melatonin (up to 3 mg) as needed were permitted for managing insomnia.
Primary Outcome Measure: The SRS-2 is a norm-referenced, 65 item, four-point Likert scale that assesses the severity of autism in children ages 4–18 years. The psychometric properties of the SRS-2 have been well established as a valid measure to assess for autistic traits.[6,7] The SRS-2 Social Communication and Interaction (SCI) scale assess social functioning across four domains including awareness, cognition, motivation, and communication. The SRS-2

© 2025 Joshi G et al. *JAMA Network Open*.

Restricted Interests and Repetitive Behavior (RRB) scale measures repetitive movements and restricted behavior, interests, and activities. The CGI scale is a clinician-rated measure for assessing overall severity and improvement (CGI-I) that was administered in this trial for assessing severity and improvement for the core features of ASD (ASD-CGI).

Safety and Tolerability Assessments: Safety and tolerability of memantine was monitored by administering a complete physical examination, battery of blood tests (complete blood count, electrolytes, glucose level, liver function test, and thyroid function test), urine tests (drug screen and pregnancy screen [females of child-bearing potential only]), and electrocardiogram at baseline and endpoint of the trial.

In the trial, the reliability of the ratings was ensured through formal training of trial investigators in the administration of outcome measures, which included inter-rater calibration. Weekly meetings were conducted with the investigators to reinforce adherence to protocol procedures, including the consistent and accurate completion of all rating scales. Additionally, each study participant was followed by the same clinician throughout the study period, ensuring intra-rater consistency in assessments. A total of five clinical investigators participated in the trial, all of whom were board-certified, highly trained paediatric psychopharmacology researchers with expertise in autism.

The ASD-CGI-Severity ratings were determined by the investigators, who were blinded to informant-rated measures, including the SRS-2. Ratings were based on the investigator's clinical judgment, informed by interviews with the participant and informant(s), and anchored to the Diagnostic and Statistical Manual of Mental Disorders, Fifth Edition–defined core features and severity level criteria for ASD.

Neuroimaging Methods

Exploratory Outcomes Measures: The proton spectra were acquired by applying a two-dimensional J-resolved (2D-JPRESS) proton magnetic resonance spectroscopy (^{1}HMRS) protocol. This sequence was selected to enhance the reliability of spectral fitting for metabolites,[8-10] allow for the isolated analysis of glutamate, and confirm that observed metabolic differences result from actual variations in metabolic levels rather than differences in T2 relaxation times.

Data Acquisition: Proton spectra were acquired at 4T using a 2D-JPRESS ^{1}HMRS protocol. An 8 cc (2 x 2 x 2 cm) single voxel was placed in the pregenual anterior cingulate cortex (*pg*ACC) along the midline such that the inferior edge of the voxel was parallel to the descending surface of the corpus callosum, as determined by T1-weighted structural MRI images used also for tissue segmentation. Data were collected in 12 TE-stepped spectra with the echo-time ranging from 30 to 250 ms in 20 ms increments, with TR = 2s, averages = 16, scan duration= 7 minutes.

Data Processing: Spectral analysis was conducted in a fully automated fashion using the commercially available LCModel package (version 6.2-1F). After the 2D-JPRESS dataset was resolved into a series of one-dimensional spectra where each spectrum was modeled and fitted with GAMMA simulated[11] J-resolved basis sets, glutamate levels were derived from the total integral across the J-series. Glutamate transverse relaxation time (T2) was obtained from the raw peak vs. TE decay curve and data quality was assessed by LCModel calculated Full Width Half Maximum (FWHM), Signal to Noise Ratio (SNR) and Cramer-Rao Lower Bounds (CRLB). In addition, each spectrum was visually inspected to ensure the absence of artifacts. To account for partial volume effects, corrections were applied following published guidelines for LCModel for gray matter, white matter, and cerebrospinal fluid percentages that were obtained by T1-weigthed structural MRI images segmentation and positional coordinates embedded in the raw spectra. To further ensure good data quality, we excluded measurements from further analyses when CRLB estimates exceeded 20%. Additionally, the potential effects of SNR, T2, FWHM, and tissue composition were compared to confirm that they did not influence glutamate measurements.

Statistical Methods

The mixed-effects regression models used to analyze the secondary measures of efficacy predicted the outcome measure from treatment group (memantine versus placebo), study visit (continuous), and the treatment group-by-study visit interaction, which is our test of efficacy. To examine the moderating effect of the *pg*ACC glutamate levels on the response to treatment, we predicted outcomes measures from treatment group, study visit (continuous), *pg*ACC glutamate levels (continuous), treatment group-by-study visit interaction, glutamate level-by-study visit interaction, treatment group-by-glutamate levels interaction, and treatment group-by-glutamate level-by-study visit interaction. All mixed-effects regression models had random intercepts by subject ID, used robust standard errors to account for the repeated measures on each subject, and used an independent covariance structure for the random effects. Our method of analysis did not impute data but rather used all available longitudinal data for participants in the analysis set provided they had data at baseline and at least one follow-up visit. All missing data were assumed to be missing at random.

We performed receiver operating characteristic (ROC) curve analysis to examine the ability of the *pg*ACC glutamate levels to identify those who did and did not respond to treatment. We performed separate ROC curve analyses in the

© 2025 Joshi G et al. *JAMA Network Open*.

memantine and placebo groups and compared their area under the curve (AUC) statistics. ROC analysis uses each value across the entire range of the *pg*ACC glutamate levels as the cutoff for defining a treatment responder and compares this classification to the “true” response classification, as defined by a ≥25% reduction in SRS-2 total raw-score and ASD-CGI-I ≤2. The ROC analysis then plots the false positive rate (1-specificity) and the true positive rate (sensitivity) for each *pg*ACC glutamate level on the x- and y-axis, respectively, to create the ROC curve. ROC analysis summarizes predictive utility with the AUC statistic. An AUC of 0.5 means that the *pg*ACC glutamate levels do not predict treatment response in any way and an AUC of 1.0 means that the *pg*ACC glutamate levels predict the treatment response perfectly. Based on the information from the ROC curve analysis, we used the Liu approach[12] to calculate the optimal cut-point to identify those participants who did and did not respond to treatment in both the memantine and placebo groups. This approach defines the optimal cut-point as the point where the product of the sensitivity and specificity is maximized. We used conditional probabilities to examine the predictive utility of these cutoff points. For each cutoff, we calculated sensitivity, specificity, the positive predictive value, negative predictive value, and the percent correctly classified.

Effect sizes are presented as standardized mean differences (SMD) for continuous outcomes and odds ratios (OR) for dichotomous outcomes. The SMDs for the difference of the changes in scores over time between memantine and placebo were calculated as Cohen’s *d* using the following calculations: the difference in the change scores between the two groups divided by the pooled standard deviation. Positive SMDs and ORs>1 indicate better improvement in the memantine group. For comparisons of ASD versus HCs, positive SMDs indicate lower glutamate levels in the ASD group. As defined by Cohen,[13] a SMD=.2 is interpreted as a small effect size, SMD=.5 as medium, SMD=.8 as large, and SMD=1.2 as very large. We considered ORs >2 to be clinically meaningful.

eResults.

Among the 37 participants with usable baseline ^{1}HMRS data, 19 were in the memantine group and 18 were in the placebo group.

<u>Neuro-imaging Tissue Composition, Data Quality, and Reproducibility</u>

Literature based morphological differences documented in individuals with ASD compared to HCs were echoed in the tissue percentage differences observed in this study between the ASD and HC participants. We corrected glutamate values accordingly in our analysis taking into account the voxel composition, thus tissue composition did not drive the findings.

Key quality metrics demonstrated good data quality: mean linewidth (FWHM) 0.07 ±0.02 ppm, SNR 14.3 ±3.1, and glutamate CRLB values <10.

To assess reproducibility, a subset of HC participants (N=13) underwent two repeated ^{1}HMRS scans of the *pg*ACC using consistent J-resolved acquisition and quantification protocol, with an average interval of 94.6 days (approximately 3 months) between scans. Glutamate levels demonstrated high test–retest reliability, with an intraclass correlation coefficient (ICC[2,1]) of 0.970 (95% CI: 0.898–0.990).

© 2025 Joshi G et al. *JAMA Network Open*.

eReferences.

1. Wechsler D. Wechsler Abbreviated Scale of Intelligence, Second Edition (WASI-II). San Antonio (TX): Pearson; 2011.
2. Lord C, Rutter M, Le Couteur A. Autism Diagnostic Interview-Revised: a revised version of a diagnostic interview for caregivers of individuals with possible pervasive developmental disorders. J Autism Dev Disord. 1994;24(5):659-685. doi:10.1007/BF02172145
3. Lord C, Rutter M, DiLavore PC, Risi S, Gotham K, Bishop S. Autism diagnostic observation schedule, second edition (ADOS-2). Torrance, CA: Western Psychological Services; 2012.
4. Orvaschel, H. Kiddie-Schedule for Affective Disorders and Schizophrenia-Epidemiologic Version (K-SADS-E). Fort Lauderdale, FL: Nova Southeastern University; 1995.
5. Petersen AC, Crockett L, Richards M, Boxer A. A self-report measure of pubertal status: Reliability, validity, and initial norms. J Youth Adolesc. 1988;17(2):117-133. doi:10.1007/BF01537962
6. Constantino JN, Davis SA, Todd RD, et al. Validation of a brief quantitative measure of autistic traits: comparison of the social responsiveness scale with the autism diagnostic interview-revised. J Autism Dev Disord. 2003;33(4):427-433. doi:10.1023/a:1025014929212
7. Constantino JN, Todd RD. Autistic traits in the general population: a twin study. Arch Gen Psychiatry. 2003;60(5):524-530. doi:10.1001/archpsyc.60.5.524
8. Gonenc A, Govind V, Sheriff S, Maudsley AA. Comparison of spectral fitting methods for overlapping J-coupled metabolite resonances. Magn Reson Med. 2010;64(3):623-628. doi:10.1002/mrm.22540
9. Henry ME, Lauriat TL, Shanahan M, Renshaw PF, Jensen JE. Accuracy and stability of measuring GABA, glutamate, and glutamine by proton magnetic resonance spectroscopy: a phantom study at 4 Tesla. J Magn Reson. 2011;208(2):210-218. doi:10.1016/j.jmr.2010.11.003
10. Jensen JE, Licata SC, Ongür D, et al. Quantification of J-resolved proton spectra in two-dimensions with LCModel using GAMMA-simulated basis sets at 4 Tesla. NMR Biomed. 2009;22(7):762-769. doi:10.1002/nbm.1390
11. Smith SA, Levante TO, Meier BH, and Ernst RR. Computer Simulations in Magnetic Resonance. An Object-Oriented Programming Approach. J Magn Reson A. 1994;106: 75-105.
12. Liu X. Classification accuracy and cut point selection. Stat Med. 2012;31(23):2676-2686. doi:10.1002/sim.4509
13. Cohen, J. Statistical Power Analysis for the Behavioral Sciences. 2nd ed. Hillsdale, NJ: Lawrence Erlbaum Associates; 1988.

© 2025 Joshi G et al. *JAMA Network Open*.

eTable 1. Demographics, Neurocognitive, Clinical, and Treatment Characteristics as Compared Between Participants With Complete and Incomplete Trial Data

	**Completers**	**Early Terminations**	***P*-value**
Sample size	33	09	
Memantine	16 (48)	05 (56)	1.00
Placebo	17 (52)	04 (44)	
**Demographic Profile**			
Age (years)			
Mean	13.2 ±2.6	13.3 ±2.7	.89
Range	8-18	9-17	
Pre-adolescents (8-12 years)	12 (36)	3 (33)	1.00
Adolescents (13-18 years)	21 (64)	6 (67)	
Sex (Male)	24 (73)	8 (89)	.42
Race (Caucasian)	32 (97)	7 (78)	.11
IQ (Full scale)	111.7 ±15.4	89.7 ±18.5	<.001
Tanner Stage ≥III*	24 (75) [N=32]	08 (89)	.65
Body Weight (pounds) [Range]	135.8 ± 48.2 [53-227]	130.1 ± 35.4 [82.2-175.5]	.74
Underweight**	1 (3)	0 (0)	.79
Overweight**	10 (30)	2 (22)	.49
**Autism Profile**			
ADOS-2	N=31	N=7	
ASD Diagnosis	30 (97)	7 (100)	1.00
-Autism	21 (68)	6 (86)	.65
-Autism Spectrum	9 (29)	1 (14)	.65
SRS-2 [R-score]			
SRS-Total	108.5 ±19.8	108.2 ±21.5	.97
SRS-SCI	87.8 ±16.8	86.8 ±19.2	.88
SRS-RRB	20.1 ±4.9	20.7 ±4.9	.77
ASD-CGI-S (≥4)	33 (100)	9 (100)	N/A
**Psychopathological Profile**			
Psychiatric Disorders (Lifetime) ***			
No Major Psychopathology	2 (6)	0 (0)	1.00
ADHD	26 (79)	8 (89)	.66
Multiple Anxiety Disorders (≥2)	24 (73)	8 (89)	.42
Major Depression	22 (67)	1 (11)	.006
Mania	6 (18)	1 (11)	1.00
Psychosis	2 (6)	4 (44)	.01
**Functional Profile**			
GAF	53.8 ±2.2	53.9 ±1.7	.93
**Psychopharmacotherapy Profile**	N=33	N=9	
Concomitant Pharmacotherapy	26 (79)	4 (44)	.09
Mean # of Medications [Range]	2.3 ±0.9 [1-4]	1.5 ±0.6 [1-2]	N/A
# of Medications	N=26	N=4	
1 medication	4 (15)	2 (50)	N/A
2 medications	13 (50)	2 (50)	N/A
≥3 medications	9 (35)	0 (0)	N/A
Class of Psychotropic Medications			
SSRIs	17 (65)	0 (0)	N/A

© 2025 Joshi G et al. *JAMA Network Open*.

Stimulants	12 (46)	2 (50)	N/A
Atomoxetine	3 (12)	0 (0)	N/A
Alpha-2 Agonists (Guanfacine, Clonidine)	9 (35)	0 (0)	N/A
Melatonin	6 (23)	1 (25)	N/A
Buspirone	4 (15)	1 (25)	N/A
Aripiprazole	3 (12)	0 (0)	N/A
Clonazepam	1 (4)	1 (25)	N/A
Amitriptyline	2 (8)	0 (0)	N/A
Trazodone	0 (0)	1 (25)	N/A
Diphenhydramine	1 (4)	0 (0)	N/A

Values expressed as N (%) or Mean ±Standard Deviation; N/A=Not Applicable; IQ=Intelligence Quotient; N/A=Not Applicable; *Petersen Pubertal Development Scale; **Per CDC stature-for-age and weight-for-age growth charts with 5th and 95th percentile cutoffs; ADOS-2=Autism Diagnostic Observation Schedule-Second Edition; R-score=Raw-score; ASD=Autism Spectrum Disorder; SRS-2=Social Responsiveness Scale-Second Edition; SRS-SCI=SRS-Social Communication and Interaction; SRS-RRB=SRS-Restricted Repetitive Behavior; ***Kiddie Schedule for Affective Disorders and Schizophrenia-Epidemiological Version; ADHD=Attention-Deficit/Hyperactivity Disorder; ASD-CGI-S=ASD-Clinical Global Impression-Severity; GAF=Global Assessment of Functioning; SSRIs=Selective Serotonin Reuptake Inhibitors

© 2025 Joshi G et al. *JAMA Network Open*.

eTable 2. Treatment Efficacy Response

**Secondary Efficacy Response**	**Memantine**			**Placebo**			**ES** [95% CI]	***P*-value**
	**Baseline**	**Endpoint**	**MD**	**Baseline**	**Endpoint**	**MD**		
Sample size	16	16		19	19			
Autism								
SRS-Total	114.5 ±21.3	77.0 ±22.5	-37.6 ±27.9	103.2 ±17.3	83.9 ±26.1	-19.4 ±26.4	0.67 [-0.02, 1.35]	.09
SRS-SCI	93.5 ±17.0	63.8 ±20.0	-29.8 ±22.2	83.8 ±14.4	69.1 ±21.2	-14.7 ±21.9	0.68 [-0.01, 1.36]	.08
SRS-RRB	21.1 ±5.9	13.4 ±3.8	-7.8 ±6.4	19.3 ±4.0	14.7 ±6.1	-4.5 ±5.1	0.56 [-0.12, 1.24]	.12
ABC-SW	12.5 ±8.6	5.6 ±5.4	-6.9 ±7.0	12.2 ±8.6	8.8 ±8.0	-3.3 ±5.3	0.59 [-0.09, 1.27]	.14
CY-BOCS-PDD	2.9 ±5.4	1.9 ±4.0	-0.9±2.1	2.4 ±4.2	2.3 ±4.0	-0.1±3.5	0.28 [-0.38, 0.95]	.70
Associated Psychopathology								
ADHD-RS	29.1 ±13.6	18.8 ±10.9	-10.3±10.6	24.9 ±9.8	19.5 ±11.5	-5.4±7.2	0.55 [-0.13, 1.22]	.15
CASI-ANX	14.7 ±8.6	7.4 ±5.6	-7.3±6.2	13.6 ±5.7	8.5 ±4.6	-5.1±6.6	0.34 [-0.33, 1.01]	.29
CDRS-R	31.4 ±17.1	22.8 ±7.5	-8.7±10.8	26.3 ±7.9	21.6 ±3.3	-4.6±6.4	0.47 [-0.21, 1.14]	.16
GAF [N: MEM=20, PBO=21]	53.8 ±2.5	60.3 ±5.1	6.5 ±5.7	54.0 ±1.6	59.7 ±6.1	5.7 ±5.6	0.15 [-0.46, 0.75]	.53

*Per *a priori* study defined criteria; Values expressed as N (%) or Mean ±Standard Deviation; MD=Mean Difference; ES=Effect Size (Cohen's *d*); SRS=Social Responsiveness Scale; SRS-SCI=SRS-Social Communication and Interaction subscale; SRS-RRB=SRS-Restricted Repetitive Behavior subscale; ABC-SW= Aberrant Behavior Checklist-Social Withdrawal subscale; CY-BOCS-PDD=Children's Yale-Brown Obsessive Compulsive Scale for Pervasive Developmental Disorder; ADHD-RS=Attention Deficit Hyperactivity Rating Scale; CASI-ANX=Children and Adolescent Symptom Inventory Anxiety Scale; CDRS-R=Children's Depression Rating Scale-Revised; GAF=Global Assessment of Functioning; MEM=Memantine; PBO=Placebo

© 2025 Joshi G et al. *JAMA Network Open*.

eTable 3. Treatment Response Based on Pregenual Anterior Cingulate Cortex (pgACC) Glutamate Levels

	**pgACC Glutamate Levels**								
	**HIGH**				**MEDIUM**				**Continuous Three-way Interaction***
	**MEM**	**PBO**			**MEM**	**PBO**			
	**MD/RR**	**MD/RR**	**ES** [95% CI]	***P*-value**	**MD/RR**	**MD/RR**	**ES** [95% CI]	***P*-value**	***P*-value**
Sample size	10	10			05	08			
**EFFICACY**									
≥25%⇊SRS-2+ ASD-CGI-I≤2	8 (80)	2 (20)	16 [1.79, 143.15]	.007	0 (0)	2 (25)	0.61 [0, 8.64]	.49	N/A
SRS-Total	-50.1 ±25.7	-22.9 ±26.9	1.03 [0.08, 1.96]	.06	-10.6 ±10.6	-15.5 ±28.8	-0.21 [-1.32, 0.92]	.65	.02
SRS-SCI	-39.8 ±21.1	-14.9 ±22.8	1.13 [0.17, 2.07]	.03	-9.2 ±5.3	-12.4 ±23.2	-0.17 [-1.28, 0.95]	.70	.007
SRS-RRB	-10.3 ±5.3	-5.9 ±4.7	0.88 [-0.05, 1.79]	.08	-1.8 ±5.1	-3.1 ±5.8	-0.24 [-1.35, 0.89]	.65	.19
ABC-SW	-8.5 ±7.6	-3.0 ±6.1	0.80 [-0.12, 1.70]	.16	-3.6 ±5.6	-4.5 ±4.2	-0.19 [-1.31, 0.93]	.74	.0001
CY-BOCS-PDD	-0.5 ±1.6	0.3 ±4.2	0.25 [-0.63, 1.13]	.98	-2.0 ±2.8	-0.6 ±2.8	0.49 [-0.66, 1.61]	.77	.44
ADHD-RS	-9.9 ±11.7	-3.8 ±5.1	0.68 [-0.23, 1.57]	.15	-8.2 ±7.4	-8.1 ±9.1	0.01 [-1.11, 1.13]	.99	.12
CASI-ANX	-10.3 ±5.1	-4.5 ±6.5	0.99 [0.04, 1.91]	.12	-2.4 ±5.2	-5.1 ±7.1	-0.42 [-1.54, 0.72]	.62	.58
CDRS-R	-9.8 ±11.2	-4.3 ±7.6	0.57 [-0.33, 1.46]	.23	-8.0 ±11.8	-4.9 ±5.5	0.37 [-0.76, 1.49]	.48	.57
GAF	9.1 ±5.4	5.4 ±5.3	0.69 [-0.22, 1.59]	.32	4.0 ±4.3 [N=8]	7.0 ±6.0	-0.57 [-1.57, -0.44]	.63	.19
**TOLERABILITY**	**N=10**	**N=10**			**N=8**	**N=8**			
Mean dose	19.5 ±1.6	20.0 ±0.0	-0.44 [-1.32, 0.45]	.34	18.13 ±5.3	20.0 ±0.0	-0.56 [-1.50, 0.50]	.33	N/A
Participants with AEs	7 (70)	6 (60)	1.52 [0.17, 15.02]	1.00	4 (50)	2 (25)	2.79 [0.25, 45.77]	.61	N/A
Treatment Limiting AEs	0 (0)	0 (0)	N/A	N/A	2 (25)	0 (0)	6.54 [0.27, 160.98]	.47	N/A
	N=7	N=7			N=6	N=2			
Mean # of AEs	2.4 ±1.3	2.5 ±1.8	0.06 [-1.03, 1.15]	.93	2.8 ±1.0	1.0 ±0.0	N/A	N/A	N/A

Values expressed as N (%) or Mean ±Standard Deviation; N/A=Not Applicable; *pg*ACC=*pregenual* Anterior Cingulate Cortex; *Treatment arm, wave, & pgACC glutamate activity levels; MEM=Memantine; PBO=Placebo; MD=Mean Difference; RR=Response Rate; ES=Effect Size (Cohen's *d*/Odds Ratio); ASD-CGI-I=ASD-Clinical Global Impression-Improvement; SRS-2=Social Responsiveness Scale-Second Edition; SRS-SCI=SRS-Social Communication and Interaction subscale; SRS-RRB=SRS-Restricted Repetitive Behavior subscale; ABC-SW=Aberrant Behavior Checklist-Social Withdrawal; CY-BOCS-PDD=Children's Yale-Brown Obsessive Compulsive Scale for Pervasive Developmental Disorder; ADHD-RS=Attention Deficit Hyperactivity Disorder Rating Scale; CASI-ANX=Children and Adolescent Symptom Inventory Anxiety Scale; CDRS-R=Children's Depression Rating Scale-Revised; GAF=Global Assessment of Functioning; AEs=Adverse Events

© 2025 Joshi G et al. *JAMA Network Open*.

eFigure. AUC Response to Treatment Based on the Baseline Pregenual Anterior Cingulate Cortex (pgACC) Glutamate Levels

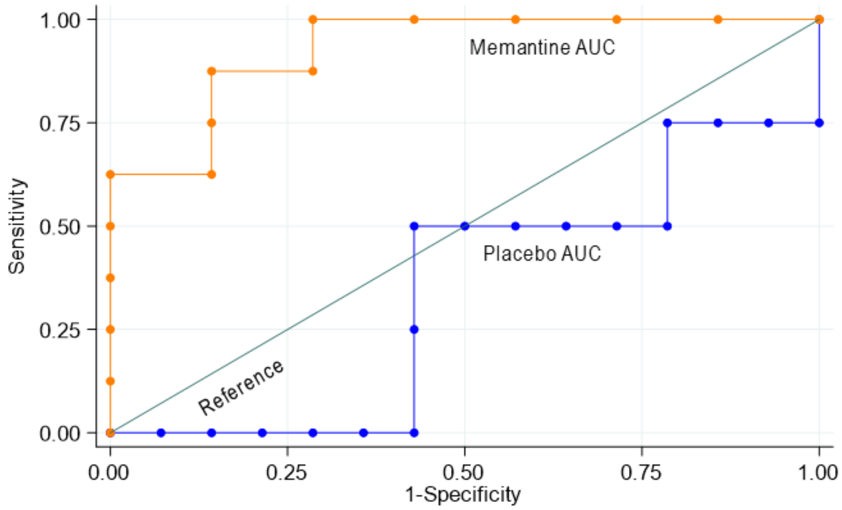

	Memantine [N=15]	Placebo [N=18]	*P*-value [t-statistic]
AUC (95% CI)	0.93 (0.80, 1.00)	0.34 (0.02, 0.66)	<.001 [x2=11.03]

	Baseline Glutamate Optimal Cut-point	Sensitivity	Specificity	PPV	NPV	% Correctly Classified
Memantine	99	88%	86%	88%	86%	87%
Placebo	97	50%	57%	25%	80%	50%

AUC=Area Under the Curve; CI=Confidence Interval; PPV=Positive Predictive Value; NPV=Negative Predictive Value

© 2025 Joshi G et al. *JAMA Network Open*.
