# Supplementary material for: Memantine to Treat Social Impairment in Youths With Autism Spectrum Disorder: A Randomized Clinical Trial
Source: JAMA Netw Open. 2025 Oct 1;8(10):e2534927. doi: 10.1001/jamanetworkopen.2025.34927 (PMC12489667; doi:10.1001/jamanetworkopen.2025.34927)
Supplement: Supplement 3. — Data Sharing Statement [file jamanetwopen-e2534927-s003.pdf]

Data Sharing Statement

Joshi. Memantine to Treat Social Impairment in Youths With Autism Spectrum Disorder. *JAMA Netw Open*. Published October 01, 2025. doi:10.1001/jamanetworkopen.2025.34927

Data
Additional Information: ClinicalTrials.gov Identifier: NCT01972074
Data available: No
